# Comparative genomics reveals diversity among xanthomonads infecting tomato and pepper

**DOI:** 10.1186/1471-2164-12-146

**Published:** 2011-03-11

**Authors:** Neha Potnis, Ksenia Krasileva, Virginia Chow, Nalvo F Almeida, Prabhu B Patil, Robert P Ryan, Molly Sharlach, Franklin Behlau, J Max Dow, MT Momol, Frank F White, James F Preston, Boris A Vinatzer, Ralf Koebnik, João C Setubal, David J Norman, Brian J Staskawicz, Jeffrey B Jones

**Affiliations:** 1 Department of Plant Pathology, University of Florida, Gainesville, FL, USA; 2 Department of Plant & Microbial Biology, University of California, Berkeley, Berkeley, CA, USA; 3 Department of Microbiology and Cell Science, University of Florida, Gainesville, FL, USA; 4 Faculdade de Computação, Universidade Federal de Mato Grosso do Sul, Campo Grande, MS, Brazil; 5 Institute of Microbial Technology (CSIR), Sector 39A, Chandigarh 160036, India; 6 BIOMERIT Research Centre, Biosciences Institute, University College Cork, Ireland; 7 Fundecitrus - Fundo de Defesa da Citricultura, Av. Adhemar Pereira de Barros, 201, 14807-040 Araraquara, SP. Brazil; 8 Department of Plant Pathology, Kansas State University, Manhattan, KS, USA; 9 Department of Plant Pathology, Physiology and Weed Sciences, Virginia Tech, Blacksburg, VA, USA; 10 Laboratoire Génome et Développement des Plantes, IRD-CNRS-Université-de Perpignan, Centre IRD, 911 Av. Agropolis, BP64501, 34394 Montpellier, France; 11 Virginia Bioinformatics Institute, Virginia Polytechnic Institute and State University, Blacksburg, VA, USA; 12 Institute of Food and Agricultural Sciences, Mid-Florida Research & Education Center, University of Florida, Apopka, FL, USA

## Abstract

**Background:**

Bacterial spot of tomato and pepper is caused by four *Xanthomonas *species and is a major plant disease in warm humid climates. The four species are distinct from each other based on physiological and molecular characteristics. The genome sequence of strain 85-10, a member of one of the species, *Xanthomonas euvesicatoria *(*Xcv*) has been previously reported. To determine the relationship of the four species at the genome level and to investigate the molecular basis of their virulence and differing host ranges, draft genomic sequences of members of the other three species were determined and compared to strain 85-10.

**Results:**

We sequenced the genomes of *X. vesicatoria *(*Xv*) strain 1111 (ATCC 35937), *X. perforans *(*Xp*) strain 91-118 and *X. gardneri *(*Xg*) strain 101 (ATCC 19865). The genomes were compared with each other and with the previously sequenced *Xcv *strain 85-10. In addition, the molecular features were predicted that may be required for pathogenicity including the type III secretion apparatus, type III effectors, other secretion systems, quorum sensing systems, adhesins, extracellular polysaccharide, and lipopolysaccharide determinants. Several novel type III effectors from *Xg *strain 101 and *Xv *strain 1111 genomes were computationally identified and their translocation was validated using a reporter gene assay. A homolog to Ax21, the elicitor of XA21-mediated resistance in rice, and a functional Ax21 sulfation system were identified in *Xcv*. Genes encoding proteins with functions mediated by type II and type IV secretion systems have also been compared, including enzymes involved in cell wall deconstruction, as contributors to pathogenicity.

**Conclusions:**

Comparative genomic analyses revealed considerable diversity among bacterial spot pathogens, providing new insights into differences and similarities that may explain the diverse nature of these strains. Genes specific to pepper pathogens, such as the O-antigen of the lipopolysaccharide cluster, and genes unique to individual strains, such as novel type III effectors and bacteriocin genes, have been identified providing new clues for our understanding of pathogen virulence, aggressiveness, and host preference. These analyses will aid in efforts towards breeding for broad and durable resistance in economically important tomato and pepper cultivars.

## Background

Bacterial spot disease of tomato and pepper presents a serious agricultural problem worldwide, leading to significant crop losses especially in regions with warm and humid climate. The disease is characterized by necrotic lesions on leaves, sepals and fruits, reducing yield and fruit quality [1]. The disease is caused by a relatively diverse set of bacterial strains within the genus *Xanthomonas*; strain nomenclature and classification for the strains that infect pepper and tomato have gone through considerable taxonomic revision in recent years. Currently, the pathogens are classified into four distinct pathogen groups (A, B, C, and D) within the genus *Xanthomonas*. Strains belonging to groups A, B and D infect both tomato and pepper. Group C strains are pathogenic only on tomato [2,3]. These phenotypically and genotypically distinct strains have different geographic distributions. Strains of group A and B are found worldwide. C strains have been increasingly found in the U.S., Mexico, Brazil, Korea and regions bordering the Indian Ocean, and D group strains are found in the former Yugoslavia, Canada, Costa Rica, U.S, Brazil and regions of the Indian Ocean [4-8]. Three of the four groups except for D were originally described as a single pathovar within *Xanthomonas campestris *and referred to as *X. campestris *pv. *vesicatoria*. The D group consisted of a strain isolated from tomato that had been designated '*Pseudomonas gardneri*' for many years [9] although De Ley provided evidence for placement in the genus *Xanthomonas *[10]. Subsequently all four groups were classified as separate species on the basis of physiological and molecular characteristics as follows: *Xanthomonas euvesicatoria *(group A), *Xanthomonas vesicatoria *(group B), *Xanthomonas perforans *(group C), and *Xanthomonas gardneri *(group D) [11].

Based on 16S rRNA analysis, *X. euvesicatoria *strain 85-10 (A group) and *X. perforans *(C group) together form a monophyletic group, whereas *X. vesicatoria *(B group) and *X. gardneri *(D group) cluster together with *X. campestris *pv. *campestris *(*Xcc*) *Xcc *strain 33913 [11]. Recently, a phylogenetic tree was constructed based on MLST (multi-locus sequence typing) data for A, B, C and D group strains and other xanthomonads [12]. The MLST approach revealed that *X. euvesicatoria *and *X. perforans *form a group along with *X. citri *strain 306 (*Xac*). *X. gardneri *is most closely related to *X. campestris *pv. *campestris *strains while *X. vesicatoria *forms a distinct clade [12]. This diversity among the four groups makes the *Xanthomonas*-tomato/pepper system an excellent example to study pathogen co-evolution, as distinct species have converged on a common host.

While integrated management approaches for control of bacterial spot disease are available, the development of host resistance is more economical and environmentally benign for the control of the disease [13,14]. Host resistance may also be required to replace the loss of some integrated management tools. Use of copper and streptomycin sprays over the years, for example, has led to the development of resistant strains [5]. At the same time, genetic resistance has been lost due to race shifts in pathogen populations [15-17]. Designing new and possibly durable resistance requires knowledge of pathogenicity factors possessed by the four groups.

Many candidate pathogenicity factors have been identified in strains of *Xanthomonas*. A number of virulence factors are employed by xanthomonads to gain entry into leaf or fruit tissue, and gain access to nutrients, while simultaneously overcoming or suppressing plant defenses. Different secretion systems and their effectors have been shown to contribute to the virulence of plant pathogens. The type III secretion system (T3SS) encoded by the *hrp *(Hypersensitive Response and Pathogenicity) gene cluster [18,19] and type III secreted effectors have been widely studied for their role in hypersensitivity and pathogenicity. Effectors common between strains are believed to be responsible for conserved virulence function and avoidance of host defense. Differences in effector suites have evolved in closely related strains of plant pathogens and strain-specific effectors may help to escape recognition by host-specific defenses [20-25]. Important insights into pathogenicity mechanisms of *X. euvesicatoria *strain 85-10 (hereafter, *Xcv*) have been obtained with its genome sequence [26]. Here we report draft genome sequences of type strains of the other three bacterial spot pathogen species: *X. vesicatoria *strain 1111 (*Xv *1111) (ATCC 35937), *X. perforans *strain 91-118 (*Xp *91-118), and *X. gardneri *strain 101 (*Xg *101) (ATCC 19865). We have annotated and analyzed predicted pathogenicity factors in the draft genomes. Additionally, we have investigated differentiation between xanthomonads that might explain differences in disease phenotypes and in host range.

## Results and Discussion

### Draft genome sequences of *Xv *strain 1111, *Xp *strain 91-118 and *Xg *strain 101 were obtained by combining Roche-454 (pyrosequencing) and Illumina GA2 (Solexa) sequencing data

Initially, we sequenced *Xv *strain 1111 (ATCC 35937) (hereafter *Xv*), *Xp *strain 91-118 (hereafter *Xp*) and *Xg *strain 101 (ATCC 19865) (hereafter *Xg*) by 454 pyrosequencing [27]. *De novo *assembly using Newbler assembler resulted in 4181, 2360 and 4540 contigs, respectively, for *Xv*, *Xp *and *Xg*, with approximately 10-fold coverage for each strain (Additional file 1: Table S1). Many pathogenicity genes, including type III effectors, existed in the form of fragments given the relatively low coverage of the 454-based assembly. More complete assemblies were obtained using Illumina sequencing [28]. *De novo *assemblies of around 100-fold coverage were constructed from the Illumina data alone or combined with pre-assembled 454 long reads using CLC Genomic Workbench [29]. Combined 454 and Illumina sequencing produced a much better assembly than either technology alone (Table 1). Therefore, combined assemblies were chosen for all subsequent analyses. The average contig size in the combined 454 and Illumina assemblies was around 18 kb for *Xv *and *Xp*, and 10 kb for *Xg*. The N50 (minimum number of contigs needed to cover 50% of the assembly) values were 37 and 40 for *Xv *and *Xp*, respectively, and 83 for *Xg *indicating that final assemblies consist of a few large contigs allowing reasonably accurate whole genome comparisons.

**Table 1:** General sequencing and combined (454 and solexa) *de novo *assembly features of draft genomes of *Xv*, *Xp *and *Xg*.

	*Xanthomonas vesicatoria *(*Xv*)	*Xanthomonas perforans *(*Xp*)	*Xanthomonas gardneri *(Xg)
Number of contigs	296	291	552
N50*	37	40	83
Mean contig length	18,686	18,082	10,014
Longest contig	153,834	133,836	88,536
Total length of contigs	5,531,090	5,262,127	5,528,125

*N50 - number of contigs that cover 50% of the genome assembly.

The three strains were deduced to contain plasmids as evidenced by the presence of genes that are known to be involved in plasmid maintenance (e.g. *parB/F *genes). We have used adjacency to such genes to infer occurrence of certain other genes on plasmids.

### Relationships of the strains to other xanthomonads using whole genome comparisons

**Figure 1:**
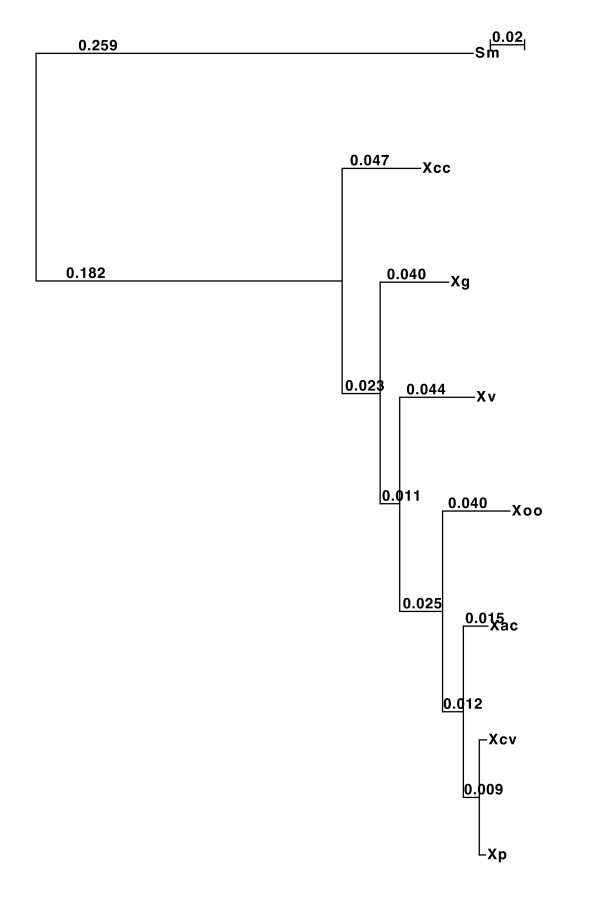
**Maximum likelihood tree based on orthologous genes from xanthomonads and *Stenotrophomonas***. Concatenated amino acid sequences of the orthologous genes from four bacterial spot pathogen strains along with other sequenced xanthomonads were considered in the analysis. *Stenotrophomonas maltophilia *(*Sm*) was used as an outgroup. The evolutionary history was inferred using the Maximum likelihood method. The tree is drawn to scale, with branch lengths corresponding to the evolutionary distances. The evolutionary distances were computed using the Maximum Composite Likelihood method and are in the units of the number of base substitutions per site.

16S rRNA analysis and MLST-based phylogenetic analysis showed the diversity among the four bacterial spot species. We carried out phylogenetic analysis based on orthologous protein-coding genes from draft genomes and reference xanthomonads (Figure 1). Whole genome comparisons were performed using the MUMi index [30] to assess pairwise distance between the draft genomes and available reference *Xanthomonas *genomes as shown in the phylogenetic tree and the distance matrix (Additional file 2: Fig. S2). Another program, dnadiff, based on nucmer [31] showed the extent of homologies among the shared regions of the genomes by pairwise comparisons (Additional file 3: Table S3). All of the methods yielded consistent results: we were able to ascertain that among the three newly sequenced strains in relationship to the previously sequenced strains, *Xp *and *Xcv *form the closest pair, which is in turn closest to *Xac*. Next, *Xg *is closest to *Xcc*, with *Xv *forming a clade with *Xg *and the *Xcc *species group (Figure 1, Additional files S2 and S3).

### Four xanthomonads show variation in the organization of the type III secretion gene clusters

**Figure 2:**
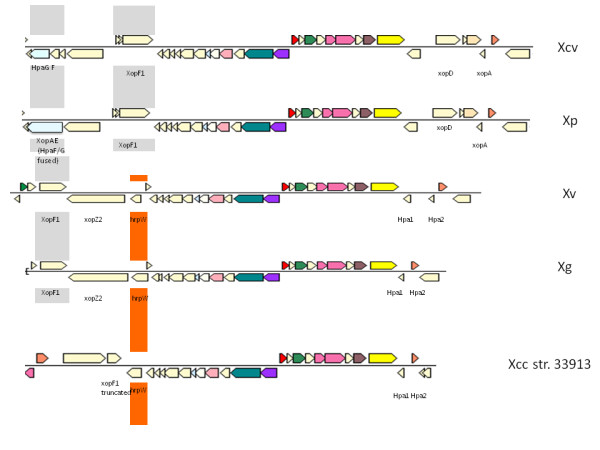
**Comparison of type III secretion system cluster, its associated type III effector genes and helper genes of three draft genomes with already sequenced xanthomonads**. Type III secretion gene clusters in five strains are shown. Boxes of the same colour indicate orthologous genes. Genes of special interest discussed in the paper are labeled. *Xp *has near identical *hrp *cluster as *Xcv*; *Xv *and *Xg *contain mosaic *hrp *cluster with organization and gene content similar to *Xcc*, but associated effectors are similar to *Xcv *along with novel effector gene associated with the cluster.

Annotation of the respective type III secretion gene clusters, or *hrp *genes showed that *Xp *has an almost identical and syntenic *hrp *cluster to that of *Xcv *(Figure 2). The most notable difference is that *hpaG *and *hpaF *encode the fusion protein XopAE in *Xp*, while they are present as separate genes in *Xcv*. Adjacent hypothetical protein XCV0410 (126 amino acid protein) is absent from *Xp*. *Xv *and *Xg *show greater similarity to the core *hrp *cluster genes of *Xcc *than to that of *Xcv*. *Xv *and *Xg *contain *hrpW *associated with the *hrp *cluster as in *Xcc*. Additionally, *xopD *in *Xv *and *Xg *is not associated with the *hrp *cluster as in *Xcc *(referred to as *psv *in *Xcc*). *PsvA *shows 74% and 84% sequence identity to the respective homologs from *Xv *and *Xg*. *XopA *(*hpa1*) from *Xcv *seems to be absent from *Xv *and *Xg*. Interestingly, we found a novel candidate effector gene (named *xopZ2*) upstream of *hrpW *in *Xv *and *Xg *(See below, Additional file 4: Fig. S4). Finally, the *hrp*-associated effector *xopF1 *is conserved and intact in all four tomato and pepper pathogens.

### A reporter gene assay confirms translocation of novel type III effectors

We identified and annotated T3SS effectors from the three newly sequenced xanthomonads (See Methods). Several candidate effectors, which had not yet been experimentally confirmed in xanthomonads, and candidate effectors with plausible translocation motifs were identified (Tables 2, 3, and 4). Corroborative evidence for T3SS-mediated translocation of the candidate effectors was assessed by constructing fusion genes with the C-terminal end of AvrBs2 coding sequence (avrBs2_62-574aa_) in a race 6 strain of *X. euvesicatoria*. Translocation was measured in pepper cv. ECW 20R, containing the resistance gene *Bs2 *(Additional file 4: Fig. S4). Genes *xopAO, xopG, xopAM*, and XGA_0724 (belonging to the *avrBs1 *class of effectors), of which homologs were previously found in *Pseudomonas *species, were demonstrated to direct AvrBs2-specific hypersensitive reactions in ECW 20R (Tables 3, Table 4, Additional file 4: Fig. S4). Another candidate effector gene *xopZ2*, associated with the *hrp *clusters in *Xv *and *Xg *(Figure 2), was also functional in the AvrBs2-based assay. Thus, we identified five effectors (*xopAO*, *xopG*, *xopAM*, *xopZ2*, XGA_0724) that have not been previously recognized in *Xanthomonas *and showed their functionality.

**Table 2:** Core effectors present in all four tomato and pepper xanthomonads

Effector class	*Xcv*	*Xv*	*Xp*	*Xg*	Pfam domains	References
AvrBs2	XCV0052	XVE_4395	XPE_2126	XGA_3805	Glycerophosphoryl diester phosphodiesterase	[104]
XopD	XCV0437	XVE_2372	XPE_2945	XGA_3151	C48-family SUMO cysteine protease (Ulp1 protease family); EAR motif	[105]
XopF1	XCV0414	XVE_3220	XPE_2922	XGA_2763	-	[105]
XopK	XCV3215	XVE_0354	XPE_1077	XGA_3563	-	[106]
XopL	XCV3220	XVE_0359	XPE_1073	XGA_0320	LRR protein	[107]
XopN	XCV2944	XVE_0564	XPE_1640	XGA_0350	ARM/HEAT repeat	[108]
XopQ	XCV4438	-	XPE_0810	XGA_0949	Inosine uridine nucleoside N-ribohydrolase	[105]
XopR	XCV0285	XVE_0593	XPE_1215, XPE_3295	XGA_1761	-	[106]
XopX	XCV0572	XVE_ 3610 XVE_3609 (partial)	XPE_1488 XPE_1553	XGA_3272 (second copy with frameshift)	-	[109]
XopZ1	XCV2059	+ (*)	XPE_2869	+(*)	-	[106]
XopAD	XCV4315/4314/4313	XVE_4177	XPE_4269	XGA_0755	SKWP repeat protein	[110]

*Xv and Xg contain effector *xopZ2 *belonging to the same family *xopZ*.

**Table 3:** Type III effectors specific to each species

Effector	Locus tags	Effector homolog	Pfam domains/biochemical motifs	Comments/Reference
**Effectors specific to *Xv***				

XopJ2	XVE_4840 (partial); XVE_3769 (partial)	AvrBsT	C55-family cysteine protease or Ser/Thr acetyltransferase	[40]
XopAG	XVE_2415	AvrGf1	-	[39]
XopAI	XVE_4756	XAC3230	-	[25]

**Effectors specific to *Xg***				

class avrBs1	XGA_0724	AvrA (84% identity)	-	This study
AvrHah1 (Fragmented in assembly)	XGA_4845/XGA_3187	AvrBs3	Transcriptional activator, nuclear localization	AvrBs3 present in few euvesicatoria strains [41].
XopAO	XGA_1250	AvrRpm1 (61% identity)	-	This study
XopAQ	XGA_2091	Rip6/rip11	No known domains	[46]
XopAS	XGA_0764/0765	HopAS1	No known domains	This study

**Effectors specific to *Xp***				

XopC2	XPE_3585	Rsp1239	Haloacid dehalogenase-like hydrolase	[24]
XopJ4	XPE_1427	AvrXv4	SUMO protease (experimental); YopJ-like serine threonine acetyl transferase domain (predicted)	[38,105]
XopAF	XPE_4185	AvrXv3	Transcriptional activator domain	[37]
XopAE	XPE_2919	HpaF/G	LRR protein	[24]

**Effectors specific to *Xcv***				

AvrBs1	XCVd0104	AvrBs1	-	[26]
XopC1	XCV2435	XopC	Phosphoribosyl transferase domain and haloacid dehalogenase-like hydrolase	[105]
XopJ1	XCV2156	XopJ	C55-family cysteine protease or Ser/Thr acetyltransferase	[105]
XopJ3	XCV0471	AvrRxv	C55-family cysteine protease or Ser/Thr acetyltransferase	[26]
XopO	XCV1055		Unknown	[26]
XopAA	XCV3785	ECF	Early chlorosis factor, proteasome/cyclosome repeat	[26]
XopAI	XCV4428	AvrRxo1	-	[26]

**Table 4:** Effectors specific to particular groups of species

Effector class	Locus tags	Pfam domains	Comments/References
**Effectors common to all pepper pathogens *Xv*, *Xcv *and *Xg***			

XopE2	XCV2280, XVE_1190, XGA_2887	Putative transglutaminase	[114]
XopG	XCV1298, XVE_4501, XGA_4777	M27 family peptidase clostridium toxin	This study

**Effectors common to *Xv*, *Xg *but absent from *Xp *and *Xcv***			

XopAM	XVE_4676, XGA_3942	-	This study
HrpW	XVE_3222, XGA_2761	Pectate lyase	HrpW associated with hrp cluster, May not be T3SE [111]
AvrXccA1	XVE_5046, XGA_0679	LbH domain containing hexapeptide repeats (X-[STAV]-X-[LIV]-[GAED]-X)- acyltransferase enzyme activity	May not be T3SE [112]
XopZ2	XGA_2762, XVE_3221	Not known	This study; Associated with hrp cluster.

**Effectors common to *Xg *and *Xcv *but absent from *Xp *and *Xv***			

XopB	XGA_4392, XCV0581	-	[113]

**Effectors common to *Xp *and *Xcv *but absent from *Xg *and *Xv***			

XopE1	XPE_1224, XCV0294	Putative transglutaminase	[114]
XopF2	XPE_1639, XCV2942	-	[105]
XopI	XPE_3711, XCV0806	F-box domain	[115]
XopP	XPE_3586, XPE_4695(Partial), XCV1236		[105]
XopV	XPE_4158, XCV0657	-	[106]
XopAK	XPE_4569, XCV3786	-	Not confirmed to be effector in Xanthomonas; Homolog of effector in *Pseudomonas*.
XopAP	XPE_1567, XCV3138	Lipase class III	45% identity to homolog in *Xp*; Homolog of rip38 from R. solanacearum RS1000 [46]

**Effectors present in *Xv *and *Xp *but absent from *Xg *and *Xcv***			

XopAR	XVE_3216, XPE_2975	-	[46]

### Core effectors among four xanthomonads give insight into infection strategies of the pathogen

Comparing the draft genome sequences of the three xanthomonads with that of *Xcv *allowed us to identify the core effectors conserved in all four strains as well as strain-specific effectors (Tables 2, 3, and 4).

At least 11 effector genes form a core set of common effectors for xanthomonads infecting tomato and pepper (Table 2). Of these 11, eight effector genes (*avrBs2, xopK, xopL, xopN, xopQ, xopR, xopX *and *xopZ*) were found to be conserved in all sequenced xanthomonads including the three draft genomes presented here with the exceptions of *X. albilineans *and *X. campestris *pv. *armoraciae*. These genes might be necessary for maintaining pathogenicity of these xanthomonads in a wide range of host plants. XopN has been reported to suppress PAMP (pathogen-associated molecular pattern)-triggered immunity by interacting with tomato TARK1 and TFT1 [32]. *XopF1 *is conserved in tomato and pepper xanthomonads. Although a homolog of *xopF1 *is found in *Xcc*, the respective gene is truncated [34]. Hence, *xopF1 *is a potential pathogenicity determinant in tomato. A *xopF1 *deletion mutant of *Xcv *did not show any difference in virulence when compared to wild type *Xcv *on the susceptible cultivar of pepper ECW, suggesting XopF1 is not the lone factor for pathogenicity of *Xcv *on pepper [33]. Another effector gene, *xopD*, is associated with the *hrp *gene cluster in *Xcv *and *Xp*. However, *xopD *appears to have translocated to another location in the genome in case of *Xg, Xv *and *Xcc *strains. XopD is annotated as "Psv virulence protein" in *Xcc *genome [34] and has been shown to be a chimeric protein sharing a C terminus with XopD from *Xcv *[35]. Although *xopD *homologs from *Xv *and *Xg *are syntenic with the *psv *gene in *Xcc*, *Xv *and *Xg *have intact full-length copies of *xopD *as in *Xcv*, indicating that the x*opD *could be another effector exclusive to the tomato pathogens and a possible pathogenicity determinant in tomato. *XopD *has been shown to enhance pathogen survival in tomato leaves by delaying symptom development [36]. Two tandem copies of *xopX *are found in *Xg*. However, one gene in *Xg *appears to be inactive due to a frameshift mutation. In *Xp*, the two copies of *xopX *are found in different locations in the genome with neighboring genes, including chaperone gene *groEL*, which is also duplicated. Orthologs of *xopZ *are also found in all four xanthomonads, with 82% identity for *Xcv *and *Xp *and 35% identity for *Xg *and *Xv*. Apart from low sequence identity in *Xv *and *Xg*, gene-specific rearrangements appear to have occurred within each ortholog. We propose that the overall low amino acid relatedness (pairwise sequence identities below 50%) of this effector in *Xv *and *Xg *warrants assigning the proteins to a new family within the *xopZ *class, named x*opZ2*, while the orthologs from *Xcv *and *Xp *belong to family of *xopZ1 *as originally described in *Xoo *and as supported by pairwise sequence identities of at least 60% (see above, Figure 2, Table 4).

### Effectors unique to *Xp *might be responsible for restricting growth on pepper

*Xp *is pathogenic only on tomato. The avirulence gene, *avrXv3*, present in *Xp*, was previously shown to elicit an hypersensitive response (HR) in pepper cv. ECW [37]. An *avrXv3 *knockout mutant of *Xp *is not virulent in pepper cv. ECW indicating that other factors are associated with host specificity. Comparing effector repertoires of the pepper pathogens *Xg*, *Xcv*, and *Xv *with *Xp *may provide clues to the factors that are responsible for reduced virulence (Table 4). Besides *avrXv3*, the only effectors present in *Xp *and absent or inactive in *Xg*, *Xv *and *Xcv *are *xopC2*, *xopAE *and *xopJ4 *(*avrXv4*) (Table 3). The gene *avrXv4 *is absent from other sequenced xanthomonads and shows gene-for-gene interaction with the *Xv4 *resistance gene from the wild tomato relative *Solanum pennellii *but does not contribute to restricted growth of *Xp *on pepper [38]. The effector *xopC2 *is a homolog of the effector *rsp1239 *from *Ralstonia solanacearum *GMI1000 and *xopAE *encodes an LRR protein with homology to the *R. solanacearum *effector PopC. Both genes, *xopC2 *and *xopAE*, are truncated in *Xcv*. Therefore, these two effectors may trigger immunity in pepper. Interestingly, *Xp *contains a paralog of *xopP*. The two copies are found next to each other in the genome and share 75% identity at the amino acid level. The second copy is next to the candidate effector *xopC2*, which is unique to *Xp *among tomato and pepper pathogens. Effectors *xopC2 *and *xopP *may both act to restrict growth in pepper. Moreover, there are at least two effectors, *xopE2 *and *xopG*, present in the pepper pathogens *Xcv*, *Xv *and *Xg *but absent from *Xp*. These effectors may be essential pathogenicity factors in pepper.

### Species-specific effectors

*Xv *possesses two unique effector genes, *xopAG *(*avrGf1*) and *xopAI *(Table 3). A phylogenetic analysis of *xopAG *showed that *xopAG *from *Xv *is closely related to *xopAG *from *X. citri A^w^*, which has been shown to be responsible for causing an HR on grapefruit [39]. XopAI is a chimeric protein, which contains a conserved myristoylation motif at its N terminus, like XopJ1. This effector class also includes the homolog *XAC3230 *from *Xac *as well as XAUB_26830 and XAUC_23780 from *X. fuscans *subsp. *aurantifolii *strains B and C, respectively [25]. The presence of transposons and phage elements in close proximity helps to explain the evolution of this novel effector in *Xac *by terminal reassortment [35]. *Xv *also contains effector gene *avrBsT*, which is responsible for the hypersensitive response on pepper. Loss of the plasmid containing *avrBsT *in *Xcv *strain 75-3 allows the strain to cause disease on pepper [40].

*Xg *contains at least two effectors, *avrHah1 *(an *avrBs3*-like effector gene) and *xopB as *does *Xcv*, and share sequence identity of 82% and 86% respectively to the corresponding effectors of *Xcv*. However, AvrHah1 appears to specify a different phenotype when compared to avrBs3 from *Xcv*. *AvrHah1 *was shown to be responsible for increased watersoaking on pepper ECW-50R and 60R, whereas *Xcv *strains carrying *avrBs3 *show a phenotype that consists of small raised fleck lesions on pepper [41]. Another effector gene, *xopB*, has a PIP box at the 5' end in *Xcv*, whereas the homolog in *Xg *does not contain a PIP box. Neighboring genes to *xopB *in the respective strains are completely different between genomes, suggesting lack of synteny between the two species in this region (Table 4). *XopB *from *Xg *is 92% identical at the amino acid level to the homolog in *Xcv*. Deletion mutants of *xopB *from *Xcv *did not show any difference in virulence, indicating it does not contribute significantly to virulence [42]. However, *xopB *may contribute to virulence in *Xg*. We also identified eight effector genes that are unique to *Xcv *(Table 3). With the exception of *xopAA *(early chlorosis factor), all of these genes belong to regions of low GC content compared to average genome GC content (64.75%): *avrBs1 *(42%), *xopC1 *(48%), *xopJ1 *(*xopJ*) (57%), *xopJ3 *(*avrRxv*) (52%), *xopO *(52%), *xopAJ *(*avrRxo1*) (51%).

### Few effectors are shared among phylogenetically related group strains

Although *Xp *and *Xcv*, and *Xv *and *Xg *form distinct phylogenetic groups (Figure 1), relatively few effectors are shared between these species. For *Xp *and *Xcv*, they share at least six effectors - *xopE1, xopF2, xopP, xopV, xopAK, xopAP*, which are absent from the other two genomes (Table 4). *Xv *and *Xg *appear to be most closely related to strains of *X. campestris *pv. *campestris*, and this relationship is reflected in the suite of effector genes. In fact, *Xg *and *Xv *share four effector genes with *Xcc*, namely, *xopAM, avrXccA1, hrpW *and *xopZ2*, with the caveat that *hrpW *and *avrXccA1 *may not function as intracellular effectors (Table 4). Furthermore, the genomic regions containing these genes are syntenic in *Xg*, *Xv *and *Xcc*.

### *X. gardneri *shows evidence of effector acquisition by horizontal gene transfer

Effector homologs of *avrA, hopAS1 *and *avrRpm1 *from *P. syringae *pv. *tomato *T1 and *P. syringae *pv. *syringae *B728a are found in *Xg *with 79%, 41% and 61% identity at the amino acid level, respectively (Table 3, Additional file 4: Fig. S4). Other *X. gardneri *strains also contain these effectors based on PCR screening (data not shown). These three effectors, XGA_0724 (belonging to *avrBs1 *class), XGA_0764/XGA_0765 (*xopAS*) and XGA_1250 (*xopAO*), are unique to *X. gardneri*. The C terminal region of XGA_0724 shows 53% identity to *avrBs1 *from *Xcv*. Hence according to the *Xanthomonas *effector nomenclature [24], XGA_0724 from *Xg *was placed under the class *avrBs1*. XGA_0764/XGA_0765 and XGA_1250 have not yet been reported to be found in xanthomonads and were assigned to new classes *xopAS *and *xopAO*. *X. gardneri *strains have been found to be associated with tomato and have a lower optimum temperature for disease development similar to that of pathovars of *Pseudomonas syringae *[43]. A high score by Alien_hunter analysis [44], along with very low GC content (45% for XGA_0724 and 48% for XGA_01250, 59% for XGA_0764/XGA_0765) and the proximity of mobile genetic elements provides evidence for horizontal gene transfer (Additional file 5: Table S5). Effector *xopAS *appears to be separated into two ORFs XGA_0764 and XGA_0765 by internal stop codon. The functionality of effector *xopAS *needs to be confirmed by *in planta *reporter gene assay. *AvrA *of *P. syringae *pv. *tomato *PT23 was shown to contribute to virulence on tomato plants [45]. Acquisition of XGA_0724 by *Xg *might have conferred increased virulence on tomato. *AvrRpm1 *from *P. syringae *pv. *syringae *possesses a myristoylation motif, which is absent from homologs in *Xg*. This modification in *Xg *might have been acquired to escape host recognition. Another candidate effector gene, *xopAQ*, in *Xg *is found 68 bps downstream of a perfect PIP box. The gene shows 65% identity at the amino acid level to *rip6/11*, a novel effector from *R. solanacearum *RS1000 [46].

### All four xanthomonads contain Ax21 coding gene but only *Xcv *contains a functional sulfation gene

The *ax21 *(activator of XA21-mediated immunity) gene is conserved among *Xanthomonas *species and is predicted to encode a type I-secreted protein that may serve as a quorum sensing signaling molecule [47]. A 17-amino acid sulfated peptide from the N-terminal region of *Xanthomonas oryzae *pv. *oryzae *(*Xoo*) Ax21 (axY^S^22) was shown to bind and activate the XA21 receptor kinase from rice, demonstrating that Ax21 is a conserved PAMP that can activate plant immune signaling [48]. The *ax21 *gene is present in *Xcv *(93% identity with *Xoo *PXO99 protein), *Xp *(94%), *Xv *(91%), and *Xg *(88%). The axY^S^22 peptide is 100% conserved in *Xcv*, *Xp *and *Xv*, while in *Xg *there is a change from leucine to isoleucine at residue 20; this is unlikely to alter the activity of the peptide, since changing this residue to alanine had no effect on recognition by XA21 [48].

Recognition of axY^S^22 by the XA21 receptor requires sulfation of tyrosine 22, which requires the putative sulfotransferase RaxST. In contrast to *ax21*, the *raxST *gene is more variable in these genomes, which is consistent with a report of sequence differences in this gene among *Xoo *strains [49]. Furthermore, in *Xp*, there is a single-nucleotide insertion at position 65, causing a frameshift mutation. The *Xv *and *Xg *genomes do not contain *raxST*; therefore, the *ax21 *gene products may be nonfunctional in these strains. These findings have implications for the further study of the role of Ax21 in quorum sensing and virulence, as well as for the usefulness of the XA21 receptor to confer resistance to xanthomonads in crop plants.

### Two type II secretion systems are conserved in all four *Xanthomonas *genomes

Most cell-wall degrading enzymes, such as cellulases, polygalacturonases, xylanases, and proteases, are secreted by a type II secretion system (T2SS). The Xps T2SS, present in all xanthomonads, has been studied for its contribution to virulence in *Xcc *and *Xoo *[50,51]. Another T2SS cluster, known as the Xcs system, is found only in certain species of *Xanthomonas*, e.g. *Xcc*, *Xac*, and *Xcv*. The Xps system secretes xylanases and proteases and is under control of *hrpG *and *hrpX *[52], indicating differential regulation. Both Xps and Xcs systems are present in all three draft genomes.

### Xanthomonads possess diverse repertoires of cell-wall degrading enzymes, which are present in diverse genomic arrangement patterns

Each species of *Xanthomonas *has its own collection of genes encoding endoxylanases, endoglucanases, and pectate lyases which contribute to cell wall deconstruction during pathogenesis. We have compared these repertoires from the three draft genomes and other xanthomonads as detailed in Table 5. The genes are designated for different families of glycosyl hydrolases (GH) and polysaccharide lyases (PL) that include the enzymes that cleave glycosidic bonds in the structural polysaccharides of plant cell walls.

**Table 5:** Repertoire of cell wall degrading enzymes in xanthomonads.

Gene name	Family	Enzymatic function	*Xp*	*Xac*	*Xcv*	*Xv*	*Xg*	*Xcc *strain 33913	*Xoo *strain KACC
**Xylanases**									

*xyn10A*	GH10	Endo-β-1,4-xylanase EC:3.2.1.8	2014	4254	4360	2337	1172	4118	4429
*xyn10B*			2016	4252	4358	-	-	-	4428
*xyn10C*			2020	4249	4355	2333	0341	4115	-
*aguA*	GH67	α-glucuronidase EC:3.2.1.139	4318	4227	4333	4712	2473	4102	4419
*xyn51A*	GH51	β-D-Arabino-furanosidase EC:3.2.1.55	0180	1286	1335	1029/1030	2303	1191	1317
*xyn5A*	GH5	Endo-β-1,4-xylanase EC:3.2.1.8	4682	0933/34 partial	0965	-	-	0857	3618

**Glucanases**									

*cel8A*	GH8	Endo-1,4-β-D glucanase	1965	3516	3641	0432	-	-	-
*cel9A*	GH9		2345	2522	2704	1327	0588	2387	-

**Pectate lyases**									

*pel1A*	PL1	Pectate lyase EC:4.2.2.2	3841	3562	3687	1933	4024	0645	0821
*pel1B*			1563	2986	3132	3512	0893	2815	-
*pel1C*			-	2373	2569	-	-	-	-
*pel3A*	PL3	Pectate lyase EC:4.2.2.2	-	2922	-	3222	2761	1219	-
*pel4A*	PL4	Rhamno-galacturonan lyase EC:4.2.2.-	1975	3505	3632	2592	4531	3377/78/79	1078
*pel9A*	PL9	Pectate lyase EC:4.2.2.2	-	-	2278	1927	1853	-	2265
*pel10A*	PL10	Pectate lyase EC:4.2.2.2	-	-	-	4069	5124	0122	-

Different cell wall degrading enzymes, such as xylanase, pectate lyase, glucanases, were compared for their repertoires among already sequenced xanthomonads including our draft genomes. Genes identified by their locus tags in the respective genomes.

Genes encoding secreted endoxylanases regulated by the x*ps *genes have been described for their contributions to virulence, including XCV0965 [52] encoding GH30 endoxyalanase. The GH30 family catalyses the cleavage of methylglucuronoxylans in the cell walls of monocots and dicots at a β-1,4-xylosidic bond penultimate to one linking the xylose residue that is substituted by an α-1,2-linked 4-O-methylglucuronate residue [53,54]. Such an enzyme secreted by *Erwinia chrysanthemi *generates oligosaccharides that are not assimilated for growth, suggesting a function in which it contributes to cell wall deconstruction for access to pectates for growth substrate [53]. It is interesting to note the orthologous genes encoding GH30 enzymes are absent in *Xg *and *Xv*, with a truncated *xyn30 *gene in *Xac*. On the basis of sequence homology, *xyn30 *genes may also contribute to virulence in *Xoo, Xcc and Xp*.

**Figure 3:**
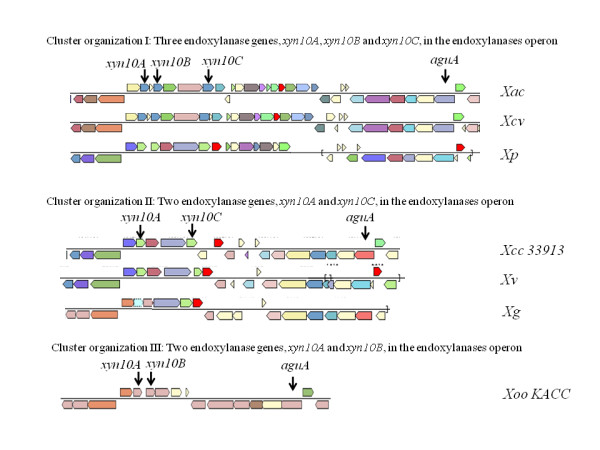
**Xylanase cluster organization**. Three types of cluster organizations can be found within xanthomonads. A) Found in *Xac, Xcv *and *Xp *containing three endoxylanase genes *xyn10A, xyn10B *and *xyn10C*; B) Found in *Xcc, Xv *and *Xg *containing two endoxylanases *xyn10A *and *xyn10C*; and C) Found in *Xoo *containing *xyn10A *and *xyn10B *within endoxylanase operon.

The more common GH10 endoxylanases, which occur in several bacterial and fungal phyla, have been implicated in the virulence of plant pathogenic bacteria and fungi [55,56]. In *Xoo*, deletion of the gene encoding a GH10 *xyn10B *resulted in diminished virulence [57]. All sequenced *Xanthomonas *genomes contain either two or three copies of *xyn10 *genes, all of which are within a gene cluster that may comprise a single operon (Figure 3). The GH10 endoxylanases are the best studied of all of the xylanases, and structure/function relationships may be inferred on the basis of gene sequence. The action of these enzymes on glucuronoxylans generates xylotriose, xylobiose, and small amounts of xylose that generally serve as substrates for growth. Also generated is methylglucuronoxylotriose, that is formed to the extent that xylose residues in the β-1,4 xylan backbone are substituted with α-1,2-linked 4-O-methylglucuronate residues [58].

An adjacent gene cluster in an opposite orientation contains *agu67 *gene encoding a GH67 α-glucuronidase that serves to catayze the removal of 4-O-methylglucuronate from the reducing terminus of methylglucuronoxylotriose. This activity provides a synergistic function to the overall xylanolytic process to generate xylotriose, which is converted to xylose by xylanases and xylosidases for complete metabolism [59]. The coregulation of operons encoding XynB and Agu67 enzymes occurs as a logical condition to coordinate expression of genes that encode these and additional enzymes that collectively process glucuronxylans and glucuronoarabinoxylans for complete metabolism. The accessory enzymes and transporters necessary for the function of these enzymes are embedded within these operons in Gram positive bacteria [60-62] and share similarities noted here with *Xanthomonas *spp.. These include the genes encoding two glycohydrolases, a β-xylosidase and an α-L-arabinofuranosidase. Also included in this cluster are genes encoding enzymes for intracellular metabolism of glucuronate and xylose, including glucuronate isomerase; xylulose isomerase; D-mannonate dehydratase; and D-mannonate oxidoreductase. Genes encoding mannitol dehydrogenase and the hexuronate transporter, as well as the TonB-dependent receptor and LacI transcriptional regulator, flank these two operons.

The arrangement and content of xylanolytic enzymes differentiate *Xanthomonas *species into three groups (Figure 3). Here, we propose a common nomenclature for xylanases, the genes for which have been annotated in the sequenced genomes. Members of the first group are *Xac*, *Xcv *and *Xp *in which all three genes encoding GH10 endoxylanases (*xyn10A*, *xyn10B *and *xyn10C*) are present, and with additional genes further downstream in this cluster. Members of the second group are *Xcc*, *Xv *and *Xg *in which genes encoding two of the three endoxylanases are present (*xyn10A *and *xyn10C*) and where one or more of the the downstream genes are absent. *Xoo *strains represent a third group in which a different set of two endoxylanase encoding genes are present (*xyn10A *and *xyn10B*) and where the β-galactosidase and gluconolactonase genes flanking *xyn10C *are absent. It is noteworthy that the organization of genes in the cluster encoding the α-glucuronidase is conserved across *Xanthomonas *species.

### Genes involved in several Type IV secretion systems are present in genomes and plasmids

**Figure 4:**
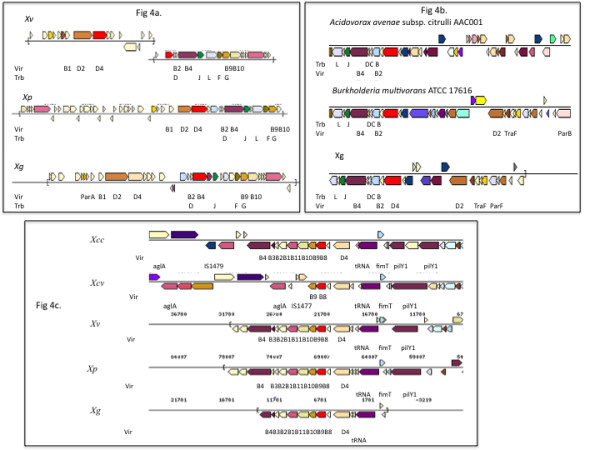
**Type IV secretion system**. A) Schematic representation of type IV secretion system cluster common to *Xp*, *Xv *and *Xg *(Plasmid borne); B) Type IV cluster unique to *Xg *(plasmid borne); C) Chromosomal type IV cluster organization in *Xcv*, *Xv*, *Xp *and *Xg*.

Like *Xcv*, the tomato pathogens, *Xg*, *Xv *and *Xp*, also appear to contain more than one copy of a type IV secretion system (T4SS) cluster (Figure 4A, B). Two T4SS clusters (Vir and Dot/Icm type) are present in *Xcv*, and genes belonging to both of these systems are found on plasmids [26]. The Dot/Icm type system is absent from *Xv*, *Xp *and *Xg*.

In *Xv *and *Xp*, genes for one T4SS are on a plasmid and the second one on the chromosome while in *Xg*, two T4SS gene clusters are on a plasmid and one is on the chromosome. The two T4SS clusters on plasmids of *Xg *do not show any similarity to the genes for T4SS in *Xac*, *Xcv*, *Xcc *and *Xoo*. Of the two T4SS clusters in *Xg*, one is also found in *Xv *and *Xp*. This cluster appears to be exclusive to these three tomato pathogens (Figure 4A). The genes belonging to this cluster show low (30-45%) identity to the T4SS clusters from *Ralstonia, Burkholderia, Bradyrhizobium*, and *Stenotrophomonas maltophilia*. The other cluster from *Xg*, which is absent from *Xv *and *Xp*, shows very high identity (98%) and synteny to the T4SS cluster of *Burkholderia multivorans *and around 89% identity to a T4SS cluster of *Acidovorax avenae *subsp. *citrulli *(Figure 4B).

Apart from the plasmid borne T4SS genes, *Xcv *also contains a portion of a type IV system cluster on the chromosome and consists of *VirB6*, *VirB8, VirB9, VirD4 *genes. This chromosomal cluster is flanked by a transposon element (IS1477) that might indicate its horizontal gene transfer. *Xp*, *Xg *and *Xv *genomes contain a complete chromosomal T4SS cluster showing high identity to the T4SS chromosomal clusters from *Xcc *(Figure 4C).

### Type V secreted adhesins function in synergism during pathogenesis

Different adhesins have been shown to function at different stages of the infection process starting with attachment, entry, later survival inside host tissue and colonization by promoting virulence [63,64]. FhaB hemagglutinin, important for leaf attachment, survival inside plant tissue and biofilm formation, is present in all four tomato pathogens. In *Xcv*, *fhaB *is divided into two separate open reading frames, XCV1860 and XCV1861, with the two-partner secretion domains being present in XCV1860. Sequence alignment indicates that *fhaB *is possibly inactivated in *Xcv *by the internal stop codon that separates XCV1860 from XCV1861. In the case of *Xoo *PXO99A, the *Xanthomonas *adhesin-like proteins XadA and XadB promote virulence by enhancing colonization of the leaf surface and leaf entry through hydathode [64]. As in *Xcv *and *Xac*, *Xp *encodes two copies of *xadA*, while *Xv *and *Xg *possess a single ortholog of *xadA *as does *Xcc*. YapH and the type IV pilus protein PilQ were shown to be involved in virulence in *Xoo *during later stages of growth and migration in xylem vessels. In *Xcv*, *Xc*, and *Xoo*KACC, two copies of *yapH *are present. There are two *pilQ *orthologs in *Xcv *and only one in other sequenced xanthomonads. Next to the *fhaB *and *fhaC *adhesin genes, *hms *operon is present in the genomes of xanthomonads, the homologs of which are *pga *operon genes in *E. coli *involved in biofilm formation [65].

### Type VI secretion system is present in *Xcv*, *Xv *and *Xp*

Type VI secretion system (T6SS) has been shown recently to contribute to host pathogen interactions during pathogenesis in *Vibrio cholerae*, *Burkholderia pseudomallei *and *Pseudomonas aeruginosa*. Hcp (Haemolysin-coregulated protein) and Vgr (valine-glycine repeats) proteins are exported by the T6SS [66]. T6SS clusters can be assigned to three different types in xanthomonads (Table 6). *Xcv *and *Xp *possess two types of T6SSs (type 1 and 3); whereas *Xv *contains only a single type of T6SS, type 3. As in *Xcc*, there is no T6SS cluster in *Xg *(Table 6, Additional file 6: Table S6).

**Table 6:** Type VI secretion clusters in different xanthomonads.

Strain	T6SS #1	T6SS #2	T6SS #3
Phosphorylation-type regulators:	Kinase/Phosphatase/Forkhead	-	Kinase/Phosphatase/Forkhead
AraC-type regulators:	-	-	AraC

*Xvv *NCPPB702	YES	/	/
*Xcm *NCPPB4381	YES	/	/
*Xaub*	/	/	YES
*Xauc*	/	/	YES
*Xac*	/	/	XAC4116 - XAC4148
*Xv*	/	/	YES
*Xp*	YES	/	YES
*Xcv*	XCV2120 - XCV2143	/	XCV4206 - XCV4244
*Xoo *KACC10331	XOO3034 - XOO3052	XOO3466 - XOO3517	/
*Xoo *MAFF 311018	XOO2886 - XOO2906	XOO3286 - XOO3319	/
*Xoo *PXO99A	XOO0245 - XOO0270	XOO2029 - XOO2060	/
*Xoc *BLS256	XOC2523 - XOC2545	XOC1309 - XOC1370	/
*Xg*	/	/	/
*Xcc *ATCC33913	/	/	/
*Xcc *8004	/	/	/
*Xca *756C	/	/	/
*Xalb*	/	/	/

### LPS locus displays remarkable variation in sequence and number of coding genes and shows host specific variation

**Figure 5:**
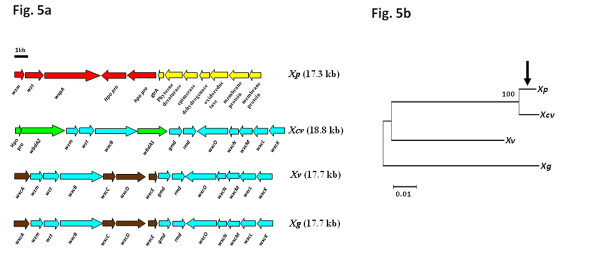
**The Structure and phylogeny of the LPS cluster**. A) Schematic comparison of LPS gene clusters described in the present study. Genes conserved in different strains are given identical color. Genes specific to individual strains are given unique color. "Hpo pro" indicates an ORF encoding for a hypothetical protein. The red color-coded genes in *Xp *genes are absent in any of the sequenced xanthomonads. B) Phylogenetic tree based on conserved *metB *and *etfA *genes that flank the variable LPS locus. Strains abbreviations are as in the main text. Arrow indicates the horizontal gene transfer event in the lineage that gave rise to *Xp*.

The lipopolysaccharide (LPS) biosynthesis cluster has been studied in detail in *Xcc *[67], which comprises three regions; region 1 from *wxcA *to *wxcE *involved in biosynthesis of water soluble LPS antigen; region 2 (*gmd, rmd*) coding for LPS core genes; and region 3 from *wxcK *to *wxcO *coding for enzymes for modification of nucleotide sugars and sugar translocation systems. This LPS biosynthesis locus is positioned between highly conserved housekeeping genes, namely cystathionine gamma lyase (*metB*) and electron transport flavoprotein (*etfA*), as reported in other xanthomonads [68]. Comparison of this cluster from draft genomes to the already sequenced xanthomonads revealed high variability in the number of genes and their sequences. *Xv *and *Xg *have an identical type of LPS gene cluster of 17.7 kb encoding 14 open reading frames (Figure 5A) which is similar in organization and sequence identity to the LPS locus from *Xcc *strains. Interestingly, *Xg *and *Xv *also contain two glycosyl transferases involved in synthesis of xylosylated polyrhamnan as seen in *Xcc *[69], in contrast to glycosyl transferases (*wbdA1, wbdA2*) involved in synthesis of polymannan in *Xcv *[26]. This suggests that basic structure of O-antigen in *Xg *and *Xv *is similar to *Xcc*. The three tomato/pepper pathogens *Xcv*, *Xv *and *Xg *have retained an ancestral type of LPS gene cluster (Figure 5A and 5B). On the other hand, *Xp *has acquired a novel LPS gene cluster during the course of evolution and is completely different in sequence and number of genes that are encoded. In *Xp*, this LPS locus is 17.3 kb long and encodes 12 ORFs, all of which are absent in the corresponding genomic region of *Xcv*, *Xv *or *Xg*. Also the first five ORFs flanking the *metB *side of the LPS locus in *Xp *(Figure 5A, ORFs colored in red) showed very low or no identity to region 1 of the LPS locus in the other xanthomonads. However, these ORFs still belong to the same Pfam families [70] that are usually present in this region, for example, ABC transporters and glycosyl transferases. The second half of the LPS cluster flanking *etfA *side encodes six ORFs, which are homologs of the LPS cluster genes from *Xac*, *Xcm *and *Xoo*. Phylogenetic insight based on conserved *metB *and *etfA *genes that flank the LPS locus suggest that the ancestor of all the *Xanthomonas *pathogens of pepper and tomato studied in this paper had the same LPS gene cluster, however putative horizontal gene transfer events at this locus have led to the acquisition of a novel LPS gene cluster in *Xp *(Figure 5B). Alien_hunter analysis also supports this acquisition with a high score showing this region to belong to an anomalous region (Additional file 5: Table S5). This event might have played a major role in changing the specificity of *Xp *towards tomato and its dominance over its relative(s) as reported previously [71], similar to variant epidemic strain of *Vibrio cholerae*, reported to be a major reason for its emergence and cholera outbreak during the 1990's in the Indian subcontinent [72]. Identity in terms of sequences and gene organization among pepper pathogens and absence of those genes from *X. perforans *and a novel LPS cluster in the tomato pathogen *X. perforans *suggest a role of this cluster in host specific variation.

### Analysis of DSF cell-cell signaling system

RpfC/RpfG are two-component signaling factors and are involved in DSF (diffusible signal factor) cell-cell signaling [73-76], known to co-ordinate virulence and biofilm gene expression. The genomes of *Xv*, *Xp*, and *Xg *carry an *rpf *(*r*egulation of *p*athogenicity *f*actors) gene cluster (Table 7) that is found in all xanthomonads and which encodes components governing the synthesis and perception of the signal molecule DSF [74,75]. The Rpf of the DSF system regulates the synthesis of virulence factors and biofilm formation and is required for the full virulence of *Xcc*, *Xac*, *Xoc*, and *Xoo *[77-81]. RpfF is responsible for the synthesis of DSF, whereas, RpfC and RpfG are implicated in DSF perception and signal transduction [73-76]. RpfC is a complex sensor kinase, whereas RpfG is a response regulator with a CheY-like receiver domain that is attached to an HD-GYP domain. HD-GYP domains act in degradation of the second messenger cyclic di-GMP [82]. In addition to genes encoding these products, *Xg *and *Xp *have *rpfH*, which encodes a membrane protein related to the sensory input domain RpfC but whose function is unknown. *Xv *contains *rpfH *but with an internal stop codon, whereas functional *rpfH *is present in *Xcv *and *Xcc*, and totally absent in *Xac *and *Xoo*.

**Table 7:** A comparison of *rpf *cluster from *rpfB *to *rpfG *found across a range of *Xanthomonas *genomes.

Gene Name	*Xcc*8004	*Xoo*	*Xcv*	*Xv*	*Xp*	*Xg*
*rpfB*	XC_2331	XOO2868	XCV1921	2934	0530	2948
*rpfF*	XC_2332	XOO2869	XCV1920	2932	0528	2950
*rpfC*	XC_2333	XOO2870	XCV1919	2930	0526	2952
*rpfH*	XC_2334	Absent	XCV1918	2928/2926*	0524	2954
*rpfG*	XC_2335	XOO2871	XCV1917	2924	0522	2956

### Cyclic di-GMP signaling

Cyclic di-GMP is a second messenger known to regulate a range of functions in diverse bacteria, including the virulence of animal and plant pathogens [83-85]. The cellular level of cyclic di-GMP is controlled by a balance between synthesis by GGDEF domain diguanylate cyclases and degradation by HD-GYP or EAL domain phosphodiesterases. GGDEF, EAL and HD-GYP domains are largely found in combination with other signaling domains, suggesting that their activities in cyclic di-GMP turnover can be modulated by environmental cues. A number of proteins involved in cyclic di-GMP signaling have been implicated in virulence of *Xcc *[86,87]. The genome of *Xcv *encodes 3 proteins with an HD-GYP domain and 33 proteins with GGDEF and/or EAL domains. As in other *Xanthomonas *spp., the HD-GYP domain proteins are completely conserved in *Xcv*, *Xv*, *Xg *and *Xp*. There is also almost complete conservation of GGDEF/EAL domain proteins between *Xcv *and three draft genomes, although *Xv *has no ortholog of XCV1982 (Additional file 7: Table S7). In addition, the EAL domain protein (XCVd0150) encoded on a plasmid in *Xcv *is absent in the other strains.

### Copper resistance (*cop*) genes are present in *Xv *and copper homeostasis (*coh*) genes are present in all strains

Among the *Xcv*, *Xv*, *Xp *and *Xg *strains sequenced, *Xv *is the only one resistant to copper and the only strain harboring a set of plasmid borne genes, namely *copL*, *copA, copB, copM, copG, copC, copD*, and *copF *that are also present in copper resistant strains of *Xac *(unpublished data/Behlau, F. personal communication) and *S*. *maltophilia *[88]. Genes *copA *and *copB *have been previously annotated as copper resistance related genes for many different xanthomonad genomes including *Xoo*, *Xoc*, *Xcv*, *Xac *and *Xcc*. Homologs of these genes are also present in *Xv, Xg *and *Xp *and are located on the chromosome. Additionally, upstream of *copA *on the chromosome of all strains, there is an ORF that shares homology with plasmid *copL*. In contrast to what has been published, chromosomal *copA *and *copB *are not responsible for copper resistance but likely for copper homeostasis and/or tolerance. While strains harboring the plasmid-borne *cop *genes, like in *Xv*, are resistant to copper and can grow on MGY agar (manitol-glutamate yeast agar) amended with up to 400 mg L^-1 ^of copper sulfate pentahydrate, strains that have only the chromosomal *cop *genes as for *Xcv*, *Xp *and *Xg*, are sensitive to copper and can only grow on media amended up to 75 mg L^-1 ^of copper. Nucleotide sequence of plasmid *cop *genes in *Xv *are 98% similar to the ones found in *Xac *and *Stenotrophomonas*, whereas chromosomal *copLAB *from *Xv *is 83% identical to homolog ORFs in *Xcv*, *Xg *and *Xp*. When *copL*, *copA *and *copB *genes from *Xv *located on the plasmid are compared to the homologs on the chromosome of the same strain, the identity of nucleotide sequences is 27, 73, and 65%, respectively. To avoid further confusion or misinterpretation, we suggest that the nomenclature of the chromosomal *copL*, *copA *and *copB *genes in xanthomonads should be changed to *cohL*, *cohA *and *cohB*, respectively, referring to copper homeostasis genes. New nomenclature has been adopted in the annotation of the draft genomes.

### Genes unique to *X. perforans *as compared to pepper pathogens give clues to its predominance over *Xcv *in the field and host specificity

Thirteen gene clusters were found to be specific to the tomato pathogen *Xp *when compared to the other three strains (Additional file 8: Table S8). A part of the clusters are syntenic to the genomic regions specific to the three pepper pathogens, suggesting the replacement of these genomic regions from pepper pathogens in correspond to these region in *Xp*. These replaced regions in *Xp *might provide potential candidates for host range determinants. Most notable among these regions was the LPS cluster genes (See above). Other such regions include the avirulence genes *avrXv3 *and *avrXv4*, a TIR-like domain containing protein, oxidoreductases, and bacteriocin-like proteins that were not found in any other sequenced xanthomonads. Importance of bacteriocin-like genes in *Xp *has already been studied for its predominance in the field over T1 strains [89,90]. Alien_hunter analysis showed that the bacteriocin BCN-A region belongs to an anomalous region indicating possible horizontal gene transfer of this region (Additional file 5: Table S5).

### Pepper pathogenicity/aggressiveness factors increased *in planta *growth of *Xp*

**Figure 6:**
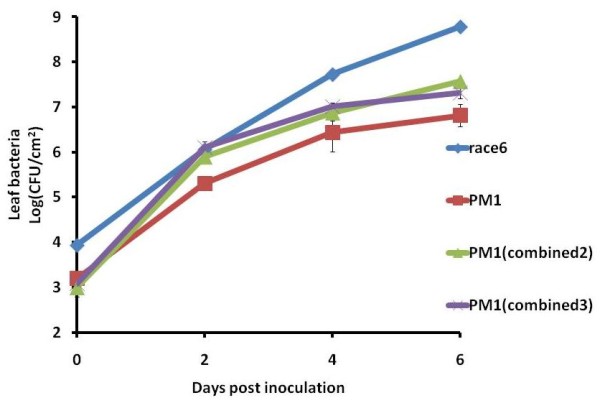
**Pepper specificity genes increasing *in planta *growth of *Xp***. *In planta *growth of PM1 transconjugants (combined 2 [XCV1839+*wxcO*]; combined 3 [XCV1839+*wxcO*+*xopG*]); PM1 and pepper virulent strain pepper race 6 represented in log (CFU/cm^2 ^of leaf tissue) at 0, 2, 4, and 6 days post inoculation.

Comparison of proteomes of *Xv, Xg, Xcv *against *Xp *showed 68 genes exclusive to pepper pathogens which might be candidate virulence factors on pepper (Additional file 9: Table S9). These include 16 genes with known function, 35 coding for mobile genetic elements, and 17 genes with unknown function/hypothetical proteins. Out of the 16 genes with known function, *xopG *was confirmed to be a type III effector using the *avrBs2 *reporter gene assay and 6 genes belong to the LPS biosynthesis gene cluster. These 16 genes were searched against already sequenced genomes of *Xac*, *Xcc *and *Xoo*. The *wxcO *gene, which codes for O-antigen, has been identified to be a virulence factor in the *X. fuscans *- bean pathosystem by subtractive hybridization [91]. Three genes, XCV1298, XCV1839 and *wxcO*, were initially selected for the verification of their contribution to virulence in pepper. Individual genes along with their promoter regions were cloned into pLAFR3 and conjugated individually and in combination into *X. perforans *ME24 (91-118Δ*avrXv3*), which no longer elicits an HR in pepper. However, *in planta *growth of ME24 is more similar to that of an avirulent strain than the virulent pepper strain TED3 race 6. ME24 transconjugants carrying *wxcO *and *XCV1839 *in combination showed increased *in planta *growth and also comparatively increased number of lesions on pepper cv. ECW when compared to ME24 revealing that these two genes play in fact a role in pepper pathogenicity (Figure 6).

### Genes specific to *Xg *as compared to other tomato/pepper pathogens may explain its aggressive nature on tomato and pepper

Comparison of genes from *Xg *against *Xcv*, *Xp *and *Xv *genes showed the presence of 625 genes specific to *Xg *(Additional file 10: Table S10). These include four type III effectors (*avrBs1 *member, *xopAO*, *avrHah1*, *xopAQ*), twenty-one genes belonging to the unique type IV secretion system cluster and associated genes. These genes can be speculated to contribute to the aggressive nature of *Xg *strains on tomato and pepper. *Xg *also contains a unique beta xylosidase not present in any other xanthomonads. Type II secreted beta xylosidase has been studied for its role in plant cell wall digestion. Moreover, *Xg *contains XGA_3730 coding for a hemolysin-type calcium-binding repeat containing protein, a homolog of which is found in *Xylella *strains with 55% sequence identity. In *Xylella*, this gene is annotated as a member of a family of pore forming toxins/RTX toxins. Its homolog is also found in other plant pathogens (i.e. *P. syringae *pv. *syringae *B728a and *R. solanacearum *GMI1000). This protein has been described as a type I effector in *X. fastidiosa *strain temecula (PD1506) [92]. RTX toxin family members, especially of the hemolysin type, have been shown to be virulence factors in a variety of cell types in eukaryotes [93,94]. Finally, a gene XGA_0603 coding for lanthionine synthetase (lantibiotic biosynthesis) is found among these *Xg *specific genes, a homolog of which is found in *Xvm *NCPPB702. LanL enzymes in pathogenic bacteria contribute to virulence by modifying the host signaling pathways, in most cases by inactivating MAPKs [95].

### Genes common to all tomato pathogens but absent from other sequenced xanthomonads

In order to see what defines the tomato pathogens, we compared the four sequenced genomes (*Xv*, *Xp*, *Xg *and *Xcv*) to other sequenced xanthomonads. We found seven genes that were conserved in all four tomato pathogens and absent from most of other sequenced xanthomonads with the exception of *Xcm, Xvv*, *Xaub *and *Xauc*, which possess homologs for six out of these seven genes (Table 8). Only the hypothetical protein XCV2641 seems to be specific to the four tomato pathogens. This gene shows only 35% sequence identity to a gene from *Xvv *and *Xcm*. A homolog of the hypothetical protein, XCV4416 was found in *Xau*, but is absent from all other sequenced xanthomonads. Genes homologous in *Xcm *and *Xvv *include two transposase genes both belonging to the transposase 17 superfamily (XCV0615, XCV0623), XCV0041 (putative penicillin amidase fragment), XCV0111 (lignostilbene-alpha, beta dioxygenase), XCV0112 (uncharacterized protein conserved in bacteria) (Table 8). Interestingly, XCV0111 encodes a protein known to be involved in phenylpropanoid degradation. Phenylpropanoids are well known plant secondary metabolites induced during defense response upon pathogen attack [96]. It appears that the four tomato pathogens along with *Xvv *and *Xcm *have acquired this function to disarm the basal plant defense.

**Table 8:** Genes present in all four tomato and pepper pathogens but absent from all other sequenced xanthomonads.

Locus tag for *Xcv*85-10	Possible function	Homolog present in any other genera	GC content
XCV0623	Transposase 17 superfamily Hypo protein -COG belonging to transposase, inactive derivatives	In *Stenotrophomonas*, *Acidovorax* *Xanthomonas campestris *pv. musacearum NCPPB4381	0.59
XCV2641	Hypothetical protein	*X. c*. musacearum and *X. c*. vasculorum (identity 37, 31% respectively)	0.65
XCV4416	Hypothetical protein	*Pectobacterium carotovorum* *X. fuscans *pv. aurantifolii	0.57
XCV0615	Transposase 17 superfamily Hypothetical protein COG1943 (transposase, inactivated derivates)	*Acidovorax*, *X. c*. musacearum and *X. c*. vasculorum	0.62
XCV0112	COG4704 uncharacterized protein conserved in bacteria	*Stenotrophomonas*, *X. c*. musacearum and *X. c*. vasculorum	0.65
XCV0111	putative lignostilbene-alpha,beta-dioxygenase-phenylpropanoid compound degradation	*Stenotrophomonas*, *Ralstonia*, *X. c*. musacearum and *X. c*. vasculorum	0.66
XCV0041	putative penicillin amidase (fragment)	*Ralstonia*, *X. c*. musacearum and *X. c*. vasculorum	0.64

### The evolution of pathogenicity clusters corresponds to the MLST-based phylogeny

**Figure 7:**
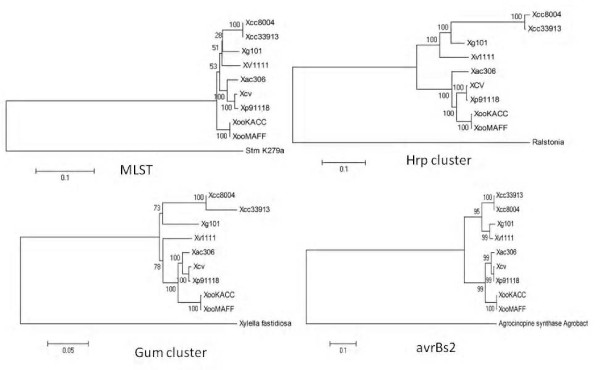
**Correlation between phylogenies based on Multi-Locus Sequence Typing (MLST) core genome and pathogenicity clusters**: Concatenated amino acid sequences of the six genes *fusA*, *gapA*, *gltA*, *gyrB*, *lacF*, *lepA *from four bacterial spot pathogen strains along with other sequenced xanthomonads are considered in the analysis. The evolutionary history was inferred using the Neighbor-Joining method. The percentage of replicate trees in which the associated taxa clustered together in the bootstrap test (1000 replicates) is shown next to the branches. The tree is drawn to scale, with branch lengths in the same units as those of the evolutionary distances used to infer the phylogenetic tree. The evolutionary distances were computed using the Maximum Composite Likelihood method and are in the units of the number of base substitutions per site. Phylogenetic analyses were conducted in MEGA4.

The correlation between tree topology using MLST and phylogeny based on the sequences of pathogenicity clusters and the *avrBs2 *effector gene, which is found in all xanthomonads, was tested. Based on MLST, *Xp *and *Xcv *group together along with *Xac *while *Xg *is more closely related to *Xcc*. *Xv *forms a different clade and is more closely related to the *Xcc *group. As can be seen in Figure 7, phylogeny based on MLST is congruent with phylogeny based on the pathogenicity clusters (*gum*, *hrp *cluster) and based on the *avrBs2 *effector, suggesting that overall these clusters were vertically inherited from the most recent common ancestor of these strains.

## Conclusions

**Figure 8:**
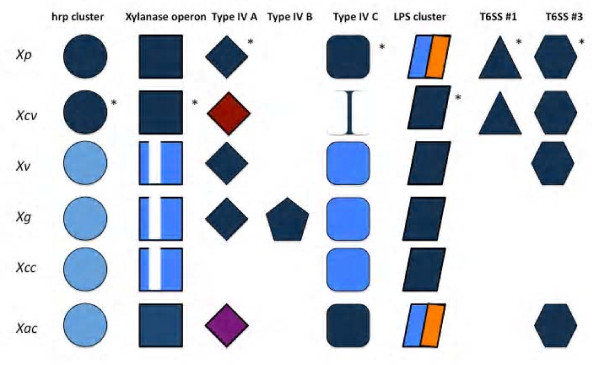
**A diagrammatic representation of relationship among bacterial spot xanthomonads, *Xac *and *Xcc *with respect to presence or absence of pathogenicity clusters**. Similar color shade indicates high identity and similar cluster organization. Lower sequence identities compared to the reference are indicated by faded gray shades. Reference strain is indicated by asterisk next to the symbol. The absence of certain part of cluster is indicated by white. In the case of LPS cluster, *Xv *and *Xac *contain novel cluster regions in the C terminal region which is indicated by a different color. *Xac *and *Xcv *contain a plasmid borne type IV cluster. Although it differs from type IVA present in other bacterial spot xanthomonads, *Xac *and *Xcv *cluster is mentioned here under type IVA with different colors. A blank space indicates complete absence of gene cluster in that particular species. A more detailed representation of individual clusters can be found in figures 2 through 5.

The interaction of *Xanthomonas *strains with tomato and pepper represents a model system for studying plant-pathogen co-evolution because of the diversity present among the strains causing bacterial spot. Although the four *Xanthomonas *species infect the same host, tomato, and cause very similar disease, they are genetically diverse pathogens. The comparative genomic analysis has provided insights into the evolution of these strains. Whole genome comparisons revealed that *Xg *and *Xv *are more closely related to *Xcc *than *Xcv *and *Xp*. A few pathogenicity clusters, such as *hrp*, *xcs *and *xps *of *Xg *and *Xv*, were similar in terms of genetic organization and sequence identity to *Xcc *(Figure 8). However, a few pathogenicity clusters of the four strains belonging to four phylogenetic groups showed different evolutionary origins. While the pepper pathogens *Xcv*, *Xv *and *Xg *possess similar LPS biosynthesis cluster, part of the LPS cluster from *Xp *is similar to the one from *Xac *(Figure 8). *Xv *contains few effectors, including *xopAG *(*avrGf1*) and *xopAI *the latter of which was previously found to be unique to citrus pathogens *Xac*, *Xaub *and *Xauc *[25]. *Xg *has a number of effectors homologous to *P*. *syringae *type III effectors suggesting probable horizontal transfer of these effectors. *Xg *contains a unique T4SS along with the one that is exclusive to *Xp*, *Xv *and *Xg*. *Xp *has two T6SSs, as found in *Xcv*. *Xv *has only one T6SS which is similar to that of *Xac*. *Xg *has no T6SS as seen for *Xcc *(Figure 8). While *Xg *and *Xv *show close relationship to *Xcc *based on whole genome comparisons, few pathogenicity clusters mentioned above seem to be conserved among tomato/pepper xanthomonads.

Type III effectors have been investigated for their contribution to pathogenicity and host-range specificity. In addition to homologs of the known effectors, we identified novel effectors in the draft genomes. By comparing effector repertoires of tomato pathogens, two possible candidate pathogenicity determinants, *xopF1 *and *xopD*, were identified, of which *xopD *is responsible for delaying symptom development, and in turn, is important for pathogen survival. Unique genes present in *Xg *include the novel effectors *xopAO*, *xopAQ, xopAS *and an *avrBs1 *member as well as a few other virulence factors, which have been characterized in other plant pathogens and which could explain the aggressive nature of *Xg *on pepper. Each species contains at least three unique type III effectors, which could explain host preferences among the strains and their aggressiveness on tomato/pepper. Comparison of the LPS clusters between the four species revealed significant variation. *Xp *has acquired a novel LPS cluster during evolution, which might be responsible for its predominance and its limited host range. As seen from the *in planta *growth assay of *Xp ΔavrXv3 *mutant carrying the LPS O-antigen from *Xcv*, the LPS cluster from pepper pathogens can be a contributor to the increased *in planta *growth of *Xp ΔavrXv3 *mutant on pepper, but is not the absolute virulence determinant. Use of the XA21 receptor similar to the *Xoo*-rice system in *Xcv *- tomato/pepper could be one of the ways to confer resistance to xanthomonads due to presence of a similar AX21 peptide and a functional rax system in *Xcv*. Common and unique genes encoding enzymes involved in cell wall deconstruction are candidates for further study to define host preference and virulence.

In conclusion, comparison of draft genomes obtained by next generation sequencing has allowed an in-depth study of diverse groups of bacterial spot pathogens at the genomic level. This analysis will serve as a basis to infer evolution of new virulent strains and overcoming existing host resistance. The knowledge of potential virulence or pathogenicity factors is expected to aid in devising effective control strategies and breeding for durable resistance in tomato and pepper cultivars.

## Methods

### Genome sequencing

*Xv*, *Xp *and *Xg *were sequenced by 454-pyrosequencing [27] at core DNA sequencing facility, ICBR, University of Florida. *Xanthomonas *isolates were grown overnight in nutrient broth. Genomic DNA was isolated using CTAB-NaCl extraction method [97] and resuspended in TE buffer (10 mM Tris pH 8, 1 mM EDTA pH 8). Libraries of fragmented genomics DNA were sequenced on 454-Genome Sequencer, FLX instrument at Interdisciplinary Center for Biotechnology Research (ICBR) at UF. *De novo *assemblies were constructed using 454 Newbler Assembler [27]. The three draft genomes were obtained with around 10× coverage.

For Illumina sequencing, the *Xanthomonas *strains were purified from single-colony and grown overnight in liquid cultures. Genomic DNA was isolated by phenol extraction and precipitated twice with isopropanol, and finally dissolved in TE buffer. DNA was then purified by cesium chloride density gradient centrifugation and precipitated with 95% ethanol, then dissolved in TE buffer. Libraries of fragmented genomic DNA with adapters for paired-end sequencing were prepared according to the protocol provided by Illumina, Inc. with minor modifications. The libraries were sequenced on the 2G Genome Analyzer at Center of Genome Research & Biocomputing at Oregon State University and post-processed using a standard Illumina pipeline [28]. We obtained approximately 8-10 million 60-bp reads for each genome, providing roughly 95× predicted coverage.

### Assembly and annotation

*De novo *assembly was generated on Newbler assembler (version 2.3; 454 Life Science, Branford, CT) using 454-sequencing reads for each genome. CLC workbench [29] was used in the next step for combining 454-based contigs with illumina reads, wherein, 454 based contigs were used as long reads to fill in gaps generated during combined *de novo *assembly. These combined assemblies of each genome were uploaded on IMG-JGI (Joint Genome Institute, Walnut Creek, California) server for gene calling. The gene prediction was carried out using GeneMark. Pfam, InterPro, COGs assignments were carried out for identified genes. Pathogenicity clusters described in the paper were manually annotated.

### Whole genome comparisons

We aligned draft genomes against reference *Xanthomonas *genomes using nucmer [31] of MUMmer program (version 3.20) and dnadiff was used to calculate percentage of aligned sequences. We have also compared genomes using the MUM index [30] to measure distances between two genomes. The maximal unique exact matches index (MUMi) distance calculation was performed using the Mummer program (version 3.20). Mummer was run on concatenated contigs or replicons (achieved by inserting a string of 20 symbols 'N' between contig or replicon sequences) of each genome. The distance calculations performed using the MUMi script are based on the number of maximal unique matches of a given minimal length shared by two genomes being compared. MUMi values vary from 0 for identical genomes to 1 for very distant genomes [30].

### Phylogenetic analysis

MLST sequences (*fusA*, *gapA*, *gltA*, *gyrB*, *lacF*, *lepA*) for all the genomes were obtained in concatenated form from PAMDB website http://pamdb.org. Genes and their corresponding amino acid sequences spanning *gum*, *hrp *cluster were downloaded from NCBI genbank sequences of sequenced genomes. Amino acid sequences of proteins of these clusters for *Xcv *and *Xcc *were used as query to search for homology against draft genomes of *Xp, Xv *and *Xg*. The amino acid sequences were then concatenated for each pathogenicity cluster and then aligned using CLUSTALW ignoring gaps. Neighbour-joining trees were constructed with boostrap value for 1000 replicates using MEGA4 [98]. Codon positions included were 1st+2nd+3rd+Noncoding. All positions containing gaps and missing data were eliminated from the dataset (Complete deletion option). There were a total of 2723 positions in the final dataset.

### Phylogeny reconstruction

*Species tree*. We used a supermatrix approach as in previous work [25]. Protein sequences of six *Xanthomonas *genomes (ingroups) and the *S. maltophilia *R551-3 genome (outgroup) were clustered in 5,096 families using OrthoMCL [99]. We then selected families with one and only one representative from each of the ingroup genomes and at most one outgroup protein, resulting in 2,282 families. Their sequences were aligned using MUSCLE [100] and the resulting alignments were concatenated. Non-informative columns were removed using Gblocks [101], resulting in 792,079 positions. RAxML [102] with the PROTGAMMAWAGF model was used to build the final tree.

### Prediction of effector repertoires, cloning of candidate effectors and confirmation using avrBs2 reporter gene assay

A database was created collecting all the known plant and animal pathogen effectors. Using all these known effectors as query, tblastn analysis was performed against all contigs of the draft genomes of *Xv*, *Xg *and *Xp *with e-value of 10^-5 ^[103]. Pfam domains were searched for possible domains found in known effectors in predicted set of ORFs of draft genome sequences. Candidate effectors were classified according to the nomenclature and classification scheme for effectors in xanthomonads recently [24]. Candidate effectors showing < 45% identity at amino acid level to the known effectors were confirmed for their translocation using *avrBs2 *reporter gene assay.

N-terminal 100 amino acid region along with upstream 500 bps sequence of candidate genes were PCR amplified using primers with BglII restriction sites at the 5' ends. Following digestion with BglII, PCR amplicons were ligated with BglII-digested pBS(BglII::*avrBs2*_62-574_::*HA*) (courtesy of Dr. Mary Beth Mudgett, Stanford university), and later transformed into *E. coli *DH5α. In-frame fusions were confirmed by DNA sequencing using F20 and R24 primers. BamHI-KpnI fragments containing the candidate gene fused to *avrBs2 *was then cloned into pUFR034. Resulting plasmids were then introduced into *Xcv *pepper race 6 (*TED3 *containing mutation in *avrBs2*) by tri-parental mating. The resulting *Xcv *strains were inoculated on *Bs2 *pepper cv. ECW 20R and kept at 28°C in growth room. After 24 hours, strong HR was indicating successful translocation of candidate effector fusions.

### Cloning of pepper specificity genes in *Xp*

The three genes mentioned above were cloned individually and in combination in pLAFR3 vector and conjugated in *Xp *91-118 Δ*avrXv3 *mutant PM1. The PM1 transconjugants with the three individual genes and combined ones along with virulent pepper race 6 strain were infiltrated at 10^5 ^CFU/ml concentration in pepper cv. ECW and leaves were sampled at every 48 hours after inoculation. The samples were plated on nutrient agar, incubated at 27°C and CFU/ml counts were enumerated. Experiment was carried out in triplicate and repeated three times.

## Database submission

The draft genome sequences of *Xanthomonas vesicatoria *ATCC 35937 (*Xv*) have been deposited at DDBJ/EMBL/GenBank under accession number AEQV00000000. The draft genome sequences of *Xanthomonas perforans *91-118 (*Xp*) have been deposited at DDBJ/EMBL/GenBank under accession number AEQW00000000. The draft genome sequences of *Xanthomonas gardneri *ATCC 19865 (*Xg*) have been deposited at DDBJ/EMBL/GenBank under accession number AEQX00000000. The version described in this paper is the first version, AEQV01000000, AEQW01000000, AEQX01000000. All three draft genomes will be released upon manuscript acceptance.

## List of abbreviations

*Xcv*: *Xanthomonas euvesicatoria *strain 85-10; formerly, *Xanthomonas campestris *pv. *vesicatoria *strain 85-10; *Xv*: *Xanthomonas vesicatoria *strain 1111 (ATCC 35937); *Xp*: *Xanthomonas perforans *strain 91-118; *Xg*: *Xanthomonas gardneri *strain 101 (ATCC 19865); *Xoo*: *Xanthomonas oryzae *pv. *Oryzae; Xcc*: *Xanthomonas campestris *pv. *Campestris; Xcm*: *Xanthomonas campestris *pv. *musacearum *NCPPB4381; *Xvv*: *Xanthomonas vasicola *pv. *vasculorum *NCPPB702; *Xac*: *Xanthomonas citri *subsp. citri strain 306; formerly, *Xanthomonas axonopodis *pv. *citri *strain 306; *Xaub*: *Xanthomonas fuscans *subsp. *aurantifolii *B strain; *Xauc*: *Xanthomonas fuscans *subsp. *aurantifolii C *strain; *Xoc: Xanthomonas oryzae pv. Oryzicola; Xca: Xanthomonas campestris *pv. *Armoracie; Xalb: Xanthomonas albilineans; Sm: Stenotrophomonas maltophilia*.

## Authors' contributions

JBJ conceived the project. JBJ and BJS oversaw genomic sequencing. JBJ provided the strains. KK did genome assembly. NP, RPR, RK, VC, PBP, FB annotated pathogenicity clusters in the genomes. DJN and BJS helped create an effector database. NP carried out effector analysis and confirmed them experimentally. NP, BAV, RK, FFW and JBJ interpreted effector analysis. NP, RPR, RK, VC, JFP, PBP, JMD, MS, TM did analyses of different pathogenicity clusters of the three genomes and helped writing corresponding sections in the manuscript. NP oversaw the experimental validations of the pathogenicity clusters. NFA created ortholog families, and did phylogenetic analysis based on all orthologus families of the three draft genomes and the reference genomes. JCS did MUMi analysis and constructed phylogenetic tree based on that analysis. NP generated the GenBank files. DJN, BJS, FFW, RK, JBJ, BAV, JFP, and JCS helped with data analyses. NP and JBJ wrote the final manuscript. All authors approved the final manuscript.

## Supplementary Material

Additional file 1 **Table S1: General features of the sequencing data and of the *de novo *assemblies of draft genomes of *Xv*, *Xp *and *Xg *using individual sequencing methods**. Click here for file

Additional file 2 **Figure S2: 1a) Phylogenetic tree based on MUMi indices; 1b) Distance matrix based on MUMi indices**. MUMi program was used to calculate pairwise distances between draft genomes and reference *Xanthomonas *genomes. Click here for file

Additional file 3 **Table S3: Whole genome comparisons using MUMmer dnadiff program**. % coverage of the aligned contigs and % identities of the respective contigs against reference genomes has been shown for each draft genome. Click here for file

Additional file 4 **Figure S4: AvrBs2-based HR assay confirms translocation of novel effectors**. Hypersensitive response reaction indicating presence of translocation signal was recorded 24 hrs after inoculation on pepper cv. ECW20R with candidate effectors *xopZ2 *(a), *avrBs1 *(b)*, xopG *(d)*, xopAM *(e)*, xopAO *(f) conjugated in race 6 strain along with control race 6 strain (c). All the strains showed water-soaking on pepper cv. ECW after 48 hrs after inoculation Click here for file

Additional file 5 **Table S5: Evidence of the horizontal gene transfer using Alien_hunter analysis**. Click here for file

Additional file 6 **Table S6: Genes/contigs representing T6SS in draft genomes as compared to *Xcv***. Click here for file

Additional file 7 **Table S7: Domain architecture and distribution of proteins with HD-GYP, GGDEF and/or EAL domains encoded by genomes of different *Xanthomonas *strains**. Click here for file

Additional file 8 **Table S8: Genes unique to *Xp*, grouped in clusters**. Click here for file

Additional file 9 **Table S9: Genes common to all pepper pathogens but absent from *Xp***. Click here for file

Additional file 10 **Table S10: Genes unique to *Xg*. Genes of special interest are highlighted in red and yellow**. Click here for file

## References

[B1] Pohronezny K Volin RB The effect of bacterial spot on yield and quality of fresh market tomatoes Hort Science 1983 18 69 70

[B2] Jones JB Stall RE Bouzar H Diversity among xanthomonads pathogenic on pepper and tomato Annu Rev Phytopathol 1998 36 41 58 10.1146/annurev.phyto.36.1.41 15012492

[B3] Jones JB Bouzar H Stall RE Almira EC Roberts PD Bowen BW Sudberry J Strickler PM Chun J Systematic analysis of xanthomonads (*Xanthomonas *spp.) associated with pepper and tomato lesions Int J Syst Evol Microbiol 2000 50 1211 1219 10843065 10.1099/00207713-50-3-1211

[B4] Bouzar H Jones JB Somodi GC Stall RE Daouzli N Lambe RC Felix Gastelum R Trinidad Correa R Diversity of *Xanthomonas campestris *pv. *vesicatoria *in tomato and pepper fields of Mexico Can J Plant Pathol 1996 18 75 77 10.1080/07060669609500659

[B5] Bouzar H Jones JB Stall RE Louws FJ Schneider M Rademaker JLW de Bruijn FJ Jackson LE Multiphasic analysis of xanthomonads causing bacterial spot disease on tomato and pepper in the Caribbean and Central America: Evidence for common lineages within and between countries Phytopathology 1999 89 328 335 10.1094/PHYTO.1999.89.4.328 18944779

[B6] Kim SH Olson TN Peffer ND Nikolaeva EV Park S Kang S First report of bacterial spot of tomato caused by *Xanthomonas gardneri *in Pennsylvania Plant Disease 2010 94 638 10.1094/PDIS-94-5-0638B 30754438

[B7] Hamza AA Robene-Soustrade I Jouen E Gagnevin L Lefeuvre P Chiroleu F Pruvost O Genetic and pathological diversity among *Xanthomonas *strains responsible for bacterial spot on tomato and pepper in the southwest Indian Ocean region Plant Disease 2010 94 993 999 10.1094/PDIS-94-8-0993 30743480

[B8] Myung IS Moon SY Jeong IH Lee YK Lee YH Ra DS Bacterial spot of tomato caused by *Xanthomonas perforans*, a new disease in Korea Plant Disease 2009 93 1349 10.1094/PDIS-93-12-1349B 30759533

[B9] Sutic D Bakterioze crvenog patlidzana (Tomato bacteriosis) Posebna Izd Inst Zasht Bilja Beograd 1957 6 1 65 (special edition). Beograd: Institute of Plant Protein. (English summary *Rev Appl Mycol *1957, **36**: 734-735.)

[B10] De Ley J Modern molecular methods in bacterial taxonomy: evaluation, application, prospects Proceedings of the 4th International Conference on Plant Pathogenic Bacteria, Angers 1978 347 357

[B11] Jones JB Lacy GH Bouzar H Stall RE Schaad NW Reclassification of the xanthomonads associated with bacterial spot disease of tomato and pepper Syst Appl Microbiol 2004 27 755 762 10.1078/0723202042369884 15612634

[B12] Almeida NF Yan S Cai R Clarke CR Morris CE Schaad NW Schuenzel EL Lacy GH Sun X Jones JB Castillo JA Bull CT Leman S Guttman DS Setubal JC Vinatzer BA PAMDB, A multilocus sequence typing and analysis database and website for plant-associated microbes Phytopathology 2010 100 208 215 10.1094/PHYTO-100-3-0208 20128693

[B13] Obradovic A Jones JB Momol MT Balogh B Olson SM Management of tomato bacterial spot in the field by foliar applications of bacteriophages and SAR inducers Plant Disease 2004 88 736 740 10.1094/PDIS.2004.88.7.736 30812485

[B14] Louws FJ Wilson M Campbell HL Cuppels DA Jones JB Shoemaker PB Sahin F Miller SA Field control of bacterial spot and bacterial speck of tomato using a plant activator Plant Disease 2001 85 481 488 10.1094/PDIS.2001.85.5.481 30823123

[B15] Kearney B Staskawicz BJ Widespread distribution and fitness contribution of *Xanthomonas campestris *avirulence gene *avrBs2* Nature 1990 346 385 386 10.1038/346385a0 2374611

[B16] Gassmann W Dahlbeck D Cjesnokova O Minsavage GV Jones JB Staskawicz BJ Molecular evolution of virulence in natural field strains of *Xanthomonas campestris *pv. *vesicatoria* J Bacteriol 2000 182 7053 7059 10.1128/JB.182.24.7053-7059.2000 11092868 PMC94833

[B17] Stall RE Jones JB Minsavage GV Durability of resistance in tomato and pepper to xanthomonads causing bacterial spot Annu Rev Phytopathol 2009 47 265 84 10.1146/annurev-phyto-080508-081752 19400644

[B18] Bonas U Schulte R Fenselau S Minsavage GV Staskawicz BJ Stall RE Isolation of a gene cluster from *Xanthomonas campestris *pv. *vesicatoria *that determines pathogenicity and hypersensitive response on pepper and tomato Mol Plant Microbe Interact 1991 4 81 88 10.1094/MPMI-4-081

[B19] Kim JG Park BK Yoo CH Jeon E Oh J Hwang I Characterization of the *Xanthomonas axonopodis *pv. glycines Hrp Pathogenicity Island J Bacteriol 2003 185 3155 3166 10.1128/JB.185.10.3155-3166.2003 12730176 PMC154065

[B20] Nimura K Melotto M He S Suppression of host defense in compatible plant- *Pseudomonas syringae *interactions Curr Opinion Plant Biol 2005 8 361 368 10.1016/j.pbi.2005.05.005 15936244

[B21] Grant SR Fisher EJ Chang JH Mole BM Dangl JL Subterfuge and manipulation: Type III effector proteins of phytopathogenic bacteria Annu Rev Microbiol 2006 60 425 449 10.1146/annurev.micro.60.080805.142251 16753033

[B22] Sarkar S Gordon J Martin G Guttman D Comparative genomics of host-specific virulence in *Pseudomonas syringae* Genetics 2006 174 1041 1056 10.1534/genetics.106.060996 16951068 PMC1602070

[B23] Rohmer L Guttman DS Dangl JL Diverse evolutionary mechanisms shape the type III effector virulence factor repertoire in the plant pathogen *Pseudomonas syringae* Genetics 2004 167 1341 1360 10.1534/genetics.103.019638 15280247 PMC1470954

[B24] White FF Potnis N Jones JB Koebnik R The Type III effectors of *Xanthomonas* Mol Plant Pathol 2009 10 749 766 10.1111/j.1364-3703.2009.00590.x 19849782 PMC6640274

[B25] Moreira LM Almeida NF Jr Potnis N Digiampietri LA Adi SS Bortolossi JC da Silva AC da Silva AM de Moraes FE de Oliveira JC de Souza RF Facincani AP Ferraz AL Ferro MI Furlan LR Gimenez DF Jones JB Kitajima EW Laia ML Leite RP Jr Nishiyama MY Neto JR Nociti LA Norman DJ Ostroski EH Pereira HA Jr Staskawicz BJ Tezza RI Ferro JA Vinatzer BA Setubal JC Novel insights into the genomic basis of citrus canker based on the genome sequences of two strains of *Xanthomonas fuscans *subsp. *aurantifolii* BMC Genomics 2010 11 238 10.1186/1471-2164-11-238 20388224 PMC2883993

[B26] Thieme F Koebnik R Bekel T Berger C Boch J Büttner D Caldana C Gaigalat L Goesmann A Kay S Kirchner O Lanz C Linke B McHardy AC Meyer F Mittenhuber G Nies DH Niesbach-Klösgen U Patschkowski T Rückert C Rupp O Schneiker S Schuster SC Vorhölter FJ Weber E Pühler A Bonas U Bartels D Kaiser O Insights into genome plasticity and pathogenicity of the plant pathogenic bacterium *Xanthomonas campestris *pv. *vesicatoria *revealed by the complete genome sequence J Bacteriol 2005 187 7254 7266 10.1128/JB.187.21.7254-7266.2005 16237009 PMC1272972

[B27] Margulies M Egholm M Altman W Attiya S Bader J Bemden L Berka J Braverman M Chen Y Chen Z Dewell S Du L Fierro J Gomes X Begley R Rothberg J Genome sequencing in open microfabricated high density picoliter reactions Nature 2005 437 376 380 16056220 10.1038/nature03959 PMC1464427

[B28] Bentley DR Whole genome resequencing Curr Opinion Genet Dev 2006 16 545 552 10.1016/j.gde.2006.10.009 17055251

[B29] CLC Genomics Workbench White paper on *de novo *assembly in CLC NGS Cell 3.0 beta 2010 http://www.clcbio.com

[B30] Delonger M Karoui ME Petit M A genomic distance based on MUM indicates discontinuity between most bacterial species and genera J Bacteriol 2009 191 91 99 10.1128/JB.01202-08 18978054 PMC2612450

[B31] Kurtz S Phillippy A Delcher AL Smoot M Shumway M Antonescu C Salzberg SL Versatile and open software for comparing large genomes Genome Biol 2004 5 R12 10.1186/gb-2004-5-2-r12 14759262 PMC395750

[B32] Kim JG Li X Roden JA Taylor KW Aakre CD Su B Lalonde S Kirik A Chen Y Baranage G McLane H Martin GB Mudgett MB *Xanthomonas *T3S effector XopN suppresses PAMP-triggered immunity and interacts with a tomato atypical receptor-like kinase and TFT1 Plant Cell 2009 21 1305 1323 10.1105/tpc.108.063123 19366901 PMC2685636

[B33] Büttner D Noel L Stutmann J Bonas U Characterization of the nonconserved *hpaB*-*hrpF *region in the *hrp *pathogenicity island from *Xanthomonas campestris *pv. *vesicatoria* Mol Plant Microbe Interact 2007 20 1063 1074 17849709 10.1094/MPMI-20-9-1063

[B34] Silva ACR Ferro JA Reinach FC Farah CS Furlan LR Quaggio R Monteiro-Vitorello CB Van Sluys MA Almeida NF Alves LMC Amaral AM Bertolini MC Camargo LE Camarotte G Cannavan F Cardozo J Chambergo F Ciapina LP Cicarelli RM Coutinho LL Cursino-Santos JR El-Dorry H Faria JB Ferreira AJ Ferreira RC Ferro MI Formighieri EF Franco MC Greggio CC Gruber A Katsuyama AM Kishi LT Leite RP Lemos EG Lemos MV Locali EC Machado MA Madeira AM Martinez-Rossi NM Martins EC Meidanis J Menck CF Miyaki CY Moon DH Moreira LM Novo MT Okura VK Oliveira MC Oliveira VR Pereira HA Rossi A Sena JA Silva C de Souza RF Spinola LA Takita MA Tamura RE Teixeira EC Tezza RI Trindade dos Santos M Truffi D Tsai SM White FF Setubal JC Kitajima JP Comparison of the genomes of two *Xanthomonas *pathogens with differing host specificities Nature 2002 417 459 463 10.1038/417459a 12024217

[B35] Stavrinides J Ma W Guttman D Terminal reassortment drives the quantum evolution of type III effectors in bacterial pathogens PLoS Pathogens 2006 2 e104 10.1371/journal.ppat.0020104 17040127 PMC1599762

[B36] Kim JG Taylor KW Hotson A Keegan M Schmelz EA Mudgett MB XopD SUMO protease affects host transcription, promotes pathogen growth, and delays symptom development in *Xanthomonas*-infected tomato leaves Plant Cell 2008 20 1915 1929 10.1105/tpc.108.058529 18664616 PMC2518228

[B37] Astua-Monge G Minsavage GV Stall RE Davis MJ Bonas U Jones JB Resistance of tomato and pepper to T3 strains of *Xanthomona campestris *pv. *vesicatoria *is specified by a plant-inducible avirulence gene Mol Plant Microbe Interact 2000 13 911 921 10.1094/MPMI.2000.13.9.911 10975648

[B38] Astua-Monge G Minsavage GV Stall RE Vallejos CE Davis MJ Jones JB *Xv4*-*Avrxv4*: A new gene-for-gene interaction identified between *Xanthomonas campetris *pv. *vesicatoria *race T3 and the wild tomato relative *Lycopersicon pennellii* Mol Plant Microbe Interact 2000 13 1346 1355 10.1094/MPMI.2000.13.12.1346 11106027

[B39] Rybak M Minsavage GV Stall RE Jones JB Identification of *Xanthomonas citri *ssp. *citri *host specificity genes in a heterologous expression host Mol Plant Pathol 2009 10 249 262 10.1111/j.1364-3703.2008.00528.x 19236573 PMC6640320

[B40] Minsavage GV Dahlbeck D Whalen MC Kearny B Bonas U Staskawicz BJ Stall RE Gene-for-gene relationships specifying disease resistance in *Xanthomonas campestris *pv. *vesicatoria*- pepper interactions Mol Plant Microbe Interact 1990 3 41 47 10.1094/MPMI-3-041

[B41] Schornack S Minsavage GV Stall RE Jones JB Lahaye T Characterization of AvrHah1, a novel AvrBs3-like effector from *Xanthomonas gardneri *with virulence and avirulence activity New Phytol 2008 179 546 556 10.1111/j.1469-8137.2008.02487.x 19086184

[B42] Noel L Thieme F Nennstiel D Bonas U C-DNA-AFLP analysis unravels a genome-wide hrpG-regulon in the plant pathogen *Xanthomonas campestris *pv. *vesicatoria* Mol Microbiol 2001 41 1271 1281 10.1046/j.1365-2958.2001.02567.x 11580833

[B43] Araujo ER Pereira RC Moita AW Ferreira MASV Café-Fiho AC Quezado-Duval AM Effect of temperature on pathogenicity components of tomato bacterial spot and competition between *Xanthomonas perforans *and *X. garnderi* III International symposium on tomato diseases 2010

[B44] Vernikos GS Parkhill J Interpolated variable order motifs for identification of horizontally acquired DNA: revisiting the *Salmonella *pathogenicity islands Bioinformatics 2006 22 2196 203 10.1093/bioinformatics/btl369 16837528

[B45] Lorang JM Shen H Kobayashi D Cooksey D Keen NT AvrA and avrE in *Pseudomonas syrinage *pv. *tomato *PT23 play a role in virulence on tomato plants Mol Plant Microbe Interact 1994 7 508 515 10.1094/MPMI-7-0508

[B46] Mukaihara T Tamura N Iwabuchi M Genome-wide identification of large repertoire of *Ralstonia solanacearum *type III effector proteins by a new functional screen Mol Plant Microbe Interact 2010 23 251 262 10.1094/MPMI-23-3-0251 20121447

[B47] Lee S.-W Jeong K.-S Han S.-W Lee S.-E Phee B.-K Hahn T.-R Ronald P The *Xanthomonas oryzae *pv. oryzae PhoPQ two-component system is required for AvrXA21 activity, *hrpG *expression, and virulence J Bacteriol 2008 190 2183 2197 10.1128/JB.01406-07 18203830 PMC2258857

[B48] Lee S.-W Han S.-W Sririyanum M Park C.-J Seo Y.-S Ronald PC A type I-secreted, sulfated peptide triggers XA21-mediated innate immunity Science 2009 326 850 853 10.1126/science.1173438 19892983

[B49] da Silva FG Shen Y Dardick C Burdman S Yadav RC de Leon AL Ronald PC Bacterial genes involved in type I secretion and sulfation are required to elicit the rice Xa21-mediated innate immune response Mol Plant Microbe Interact 2004 17 593 601 10.1094/MPMI.2004.17.6.593 15195942

[B50] Jha G Rajeshwari R Sonti R Bacterial type two secretion system secreted proteins: double-edged swords for plant pathogens Mol Plant Microbe Interact 2005 18 891 898 10.1094/MPMI-18-0891 16167759

[B51] Wang L Rong W He C Two *Xanthomonas *extracellular polygalacturonases, PghAxc and PghBxc, are regulated by type III secretion regulators HrpX and HrpG and are required for virulence Mol Plant Microbe Interact 2008 21 555 563 10.1094/MPMI-21-5-0555 18393615

[B52] Szczesny R Jordan M Schramm C Schulz S Cogez V Bonas U Büttner D Functional characterization of the Xcs and Xps type II secretion systems from the plant pathogenic bacterium *Xanthomonas campestris *pv. *vesicatoria* New Phytol 2010 187 983 1002 10.1111/j.1469-8137.2010.03312.x 20524995

[B53] Hurlbert JC Preston JF Functional characterization of a novel xylanase from corn strains of *Erwinia chrysanthemi* J Bacteriol 2001 183 2093 2100 10.1128/JB.183.6.2093-2100.2001 11222610 PMC95107

[B54] St John FJ Rice JD Preston JF Characterization of XynC from *Bacillus subtilis *subspecies *subtilis *strain 168 and Analysis of Its Role in Depolymerization of Glucuronoxylan J Bacteriol 2006 24 8617 8626 10.1128/JB.01283-06 PMC1698249 17028274

[B55] Sun Q Hu J Huang G Ge C Fang R He C Type-II secretion pathway structural gene *xpsE*, xylanase- and cellulase secretion and virulence in *Xanthomonas oryzae *pv. *oryzae* Plant Pathol 2005 54 15 21 10.1111/j.1365-3059.2004.01101.x

[B56] Goesaert H Gebruers K Brijs K Courtin CM Delcour JA XIP-type endoxylanase inhibitors in different cereals J Cereal Sci 2003 38 317 324 10.1016/S0733-5210(03)00046-8 12797742

[B57] Rajeshwari R Jha G Sonti R Role of an in planta-expressed xylanase of *Xanthomonas oryzae *pv. *oryzae *in promoting virulence on rice Mol Plant Microbe Interact 2005 18 830 837 10.1094/MPMI-18-0830 16134895

[B58] Biely P Vrsanska MM Tenkanen M Kluepfel D Endo-beta-1,4-xylanase families: differences in catalytic properties J Biotechnol 1997 57 151 166 10.1016/S0168-1656(97)00096-5 9335171

[B59] Preston JF Hurlbert JC Rice JD Ragunathan A St John FJ Mansfield SD, Saddler JN Microbial Strategies for the Depolymerization of Glucuronoxylan: Leads to the Biotechnological Applications of Endoxylanases Application of Enzymes to Lignocellulosics 2003 Ch 12 ACS Symposium Series No. 855 191 210 full_text

[B60] Shulami S Gal O Sonenshein AL Shoham Y The glucuronic acid-utilization gene cluster from *Bacillus stearothermophilus *T-6 J Bacteriol 1999 181 3695 3704 10368143 10.1128/jb.181.12.3695-3704.1999 PMC93846

[B61] Shulami S Zaide G Zolotnitsky G Langut Y Feld G Sonenshein AL Shoham Y A two-component system regulates the expression of an ABC transporter for xylo-oligosaccharides in *Geobacillus stearothermophilus* Appl Environ Microbiol 2007 73 874 84 10.1128/AEM.02367-06 17142383 PMC1800775

[B62] Chow V Nong G Preston JF Structure, function and regulation of the aldouronate-utilization gene cluster from *Paenibacillus *sp. JDR-2 J Bacteriol 2007 189 8863 8870 10.1128/JB.01141-07 17921311 PMC2168633

[B63] El Tahir Y Kuusela P Skurnik M Functional mapping of the *Yersinia enterocolitica *adhesin YadA. Identification of eight NSVAIG -S motifs on the amino-terminal half of the protein involved in collagen binding Mol Microbiol 2000 37 192 206 10.1046/j.1365-2958.2000.01992.x 10931316

[B64] Das A Rangaraj N Sonti R Multiple adhesion-like functions of *Xanthomonas oryzae *pv. *oryzae *are involved in promoting leaf attachment, entry and virulence on rice Mol Plant Microbe Interact 2009 22 73 85 10.1094/MPMI-22-1-0073 19061404

[B65] Wang X Preston JF Romeo T The *pgaABCD *locus of *Escherichia coli *promotes the synthesis of a polysaccharide adhesin required for biofilm formation J Bacteriol 2004 186 2724 2734 10.1128/JB.186.9.2724-2734.2004 15090514 PMC387819

[B66] Boyer F Fichant G Berthod J Vandenbrouck Y Attree I Dissecting the bacterial type VI secretion system by genome wide in silico analysis: what can be learned from available microbial genomic resources? BMC Genomics 2009 10 104 10.1186/1471-2164-10-104 19284603 PMC2660368

[B67] Vorhölter FJ Niehaus K Pühler A Lipopolysaccharide biosynthesis in *Xanthomonas campestris *pv. *campestris*: a cluster of 15 genes is involved in the biosynthesis of the LPS O-antigen and the LPS core Mol Genet Genomics 2001 266 79 95 11589581 10.1007/s004380100521

[B68] Patil PB Bogdanove AJ Sonti RV The role of horizontal transfer in the evolution of a highly variable lipopolysaccharide biosynthesis locus in xanthomonads that infect rice, citrus and crucifers BMC Evol Biol 2007 7 243 10.1186/1471-2148-7-243 18053269 PMC2238763

[B69] Molinaro A Silipo A Lanzetta R Newmann M Dow M Parrilli M Structural elucidation of the O-chain of the lipopolysaccharide from *Xanthomonas campestris *strain 8004 Carbohydr Res 2003 338 277 281 10.1016/S0008-6215(02)00433-0 12543561

[B70] Finn RD Mistry J Tate J Coggill P Heger A Pollington JE Gavin OL Gunesekaran P Ceric G Forslund K Holm L Sonnhammer EL Eddy SR Bateman A The Pfam protein families database Nucleic Acid Research Database issue 2010 38 D211 222 10.1093/nar/gkp985 PMC2808889 19920124

[B71] Jones JB Bouzar H Somodi GC Stall RE Pernezny K El-Morsy G Scott JW Evidence for the Preemptive Nature of Tomato Race 3 of *Xanthomonas campestris *pv. *vesicatoria *in Florida Phytopathology 1998 88 33 38 10.1094/PHYTO.1998.88.1.33 18944996

[B72] Mooi FR Bik EM The evolution of epidemic *Vibrio cholerae *strains Trends Microbiol 1997 5 161 165 10.1016/S0966-842X(96)10086-X 9141191

[B73] Slater H Alvarez-Morales A Barber CE Daniels MJ Dow JM A two-component system involving an HD-GYP domain protein links cell-cell signalling to pathogenicity gene expression in *Xanthomonas campestris* Mol Microbiol 2000 38 986 1003 10.1046/j.1365-2958.2000.02196.x 11123673

[B74] He YW Zhang LH Quorum sensing and virulence regulation in *Xanthomonas campestris* FEMS Microbiol Rev 2008 32 842 857 10.1111/j.1574-6976.2008.00120.x 18557946

[B75] Dow M Diversification of the function of cell-to-cell signaling in regulation of virulence within plant pathogenic xanthomonads Sci Signal 2008 1 23 10.1126/stke.121pe23 18506032

[B76] Ryan RP McCarthy Y Andrade M Farah CS Armitage JP Dow JM Cell-cell signal dependent dynamic interactions between HD-GYP and GGDEF domain proteins mediate virulence in *Xanthomonas campestris* Proc Natl Acad Sci USA 2010 107 5989 5994 10.1073/pnas.0912839107 20231439 PMC2851925

[B77] Barber CE Tang JL Feng JX Pan MQ Wilson TJG Slater H Dow JM Williams P and Daniels MJ A novel regulatory system required for pathogenicity of *Xanthomonas campestris *is mediated by a small diffusible signal molecule Mol Microbiol 1997 24 555 566 10.1046/j.1365-2958.1997.3721736.x 9179849

[B78] Dow JM Crossman L Findlay K He YQ Feng JX Tang JL Biofilm dispersal in *Xanthomonas campestris *is controlled by cell-cell signaling and is required for full virulence to plants Proc Natl Acad Sci USA 2003 100 10995 11000 10.1073/pnas.1833360100 12960398 PMC196915

[B79] Chatterjee S Sonti RV *rpfF *mutants of *Xanthomonas oryzae *pv. *oryzae *are deficient for virulence and growth under low iron conditions Mol Plant Microbe Interact 2002 15 463 471 10.1094/MPMI.2002.15.5.463 12036277

[B80] Siciliano F Torres P Sendín L Bermejo C Filippone P Vellice G Ramallo J Castagnaro A Analysis of the molecular basis of *Xanthomonas axonopodis *pv. *citri *pathogenesis in *Citrus limon* Electron J Biotechnol 2006 9 10.2225/vol9-issue3-fulltext-20

[B81] Wang L Makino S Subedee A Bogdanove AJ Novel candidate virulence factors in rice pathogen *Xanthomonas oryzae *pv. *oryzicola *as revealed by mutational analysis Appl Environ Microbiol 2007 73 8023 8027 10.1128/AEM.01414-07 17981946 PMC2168139

[B82] Ryan RP Fouhy Y Lucey JF Crossman LC Spiro S He YW Zhang LH Heeb S Camara M Williams P Dow JM Cell-cell signaling in *Xanthomonas campestris *involves an HD-GYP domain protein that functions in cyclic di-GMP turnover Proc Natl Acad Sci USA 2006 103 6712 6717 10.1073/pnas.0600345103 16611728 PMC1458946

[B83] Romling U Gomelsky M Galperin MY C-di-GMP: the dawning of a novel bacterial signalling system Mol Microbiol 2005 57 629 639 10.1111/j.1365-2958.2005.04697.x 16045609

[B84] Jenal U Malone J Mechanisms of Cyclic-di-GMP Signaling in Bacteria Annu Rev Genet 2006 40 385 407 10.1146/annurev.genet.40.110405.090423 16895465

[B85] Hengge R Principles of c-di-GMP signalling in bacteria Nature Rev Microbiol 2009 7 263 273 10.1038/nrmicro2109 19287449

[B86] Ryan RP Fouhy Y Lucey JF Jiang BL He YQ Feng JX Tang JL Dow JM Cyclic di-GMP signalling in the virulence and environmental adaptation of *Xanthomonas campestris* Mol Microbiol 2007 63 429 442 10.1111/j.1365-2958.2006.05531.x 17241199

[B87] He YW Boon C Zhou L Zhang LH Co-regulation of *Xanthomonas campestris *virulence by quorum sensing and a novel two-component regulatory system RavS/RavR Mol Microbiol 2009 71 1464 1476 10.1111/j.1365-2958.2009.06617.x 19220743

[B88] Crossman VC Gould JM Dow GS Vernikos A Okazaki M Sebaihia D Saunders C Arrowsmith T Carver N Peters E Adlem A Kerhornou A Lord L Murphy K Seeger R Squares S Rutter MA Quail MA Rajandream D Harris C Churcher SD Bentley J Parkhill NR Avison MB The complete genome, comparative and functional analysis of *Stenotrophomonas maltophilia *reveals an organism heavily shielded by drug resistance determinants Genome Biol 2008 9 R74 10.1186/gb-2008-9-4-r74 18419807 PMC2643945

[B89] Hert AP Roberts PD Momol MT Minsavage GV Tudor-Nelson SM Jones JB Relative importance of bacteriocin-like genes in antagonism of *Xanthomonas perforans *tomato race 3 to *Xanthomonas euvesicatoria *tomato race 1 strains Appl Environ Microbiol 2005 71 3581 3588 10.1128/AEM.71.7.3581-3588.2005 16000765 PMC1168993

[B90] Tudor-Nelson SM Minsavage GV Stall RE Jones JB Bacteriocin-like substances from tomato race 3 strains of *Xanthomonas campestris *pv. *vesicatoria* Phytopathology 2003 93 1415 1421 10.1094/PHYTO.2003.93.11.1415 18944070

[B91] Alavi SM Sanjari S Durand F Brin C Manceau C Poussier S Assessment of the genetic diversity of *Xanthomonas axonopodis *pv. *phaseoli *and *Xanthomonas fuscans *subsp. fuscans as a basis to identify putative pathogenicity genes and a type III secretion system of the SPI-1 family by multiple suppression subtractive hybridizations Appl Environ Microbiol 2008 74 3295 3301 10.1128/AEM.02507-07 18359831 PMC2394934

[B92] Reddy JD Reddy SL Hopkins DL Gabriel DW TolC is required for pathogenicity of *Xylella fastidiosa *in *Vitis vinifera *grapevines Mol Plant Microbe Interact 2007 20 403 410 10.1094/MPMI-20-4-0403 17427810

[B93] Lally ET Hill RB Kieba LR Korstoff J The interaction between RTX toxins and target cells Trends Microbiol 1999 7 356 361 10.1016/S0966-842X(99)01530-9 10470043

[B94] Linhartova I Bumba L Masin J Basler M Osicka R Kamanova J Prochazkova K Adkin I Hejnova-Holubova J Sadilkova L Morova J Sebo P RTX-toxins: a highly diverse family secreted by a common mechanism FEMS Microbiol Rev 2010 34 1076 1112 20528947 10.1111/j.1574-6976.2010.00231.x PMC3034196

[B95] Goto Y Li B Claesen J Shi Y Bibb MJ van der Donk WA Discovery of unique lanthionine synthetases reveals new mechanistic and evolutionary insights PLoS Biol 2010 8 e1000339 10.1371/journal.pbio.1000339 20351769 PMC2843593

[B96] Dixon RA Achnine L Kota P Liu C Reddy MS Wang L The phenylpropanoid pathway and plant defence - a genomics perspective Mol Plant Pathol 2002 3 371 390 10.1046/j.1364-3703.2002.00131.x 20569344

[B97] Ausubel FM Brent R Kingston RE Moore DD Seidman JG Smith JA Struhl K Current protocols in molecular biology 1994 1 New York, N. Y.: John Wiley and Sons 2.4.1 2.4.2

[B98] Tamura K Dudley J Nei M Kumar S MEGA4: Molecular Evolutionary Genetics Analysis (MEGA) software version 4.0 Mol Biol Evol 2007 24 1596 1599 10.1093/molbev/msm092 17488738

[B99] Li L Stoeckert CJ Jr Roos DS OrthoMCL: identification of ortholog groups for eukaryotic genomes Genome Res 2003 13 2178 2189 10.1101/gr.1224503 12952885 PMC403725

[B100] Edgar RC MUSCLE: multiple sequence alignment with high accuracy and high throughput Nucleic Acids Res 2004 32 1792 1797 10.1093/nar/gkh340 15034147 PMC390337

[B101] Castresana J Selection of conserved blocks from multiple alignments for their use in phylogenetic analysis Mol Biol Evol 2000 17 540 552 10742046 10.1093/oxfordjournals.molbev.a026334

[B102] Stamatakis A RAxML-VI-HPC: maximum likelihood-based phylogenetic analyses with thousands of taxa and mixed models Bioinformatics 2006 22 2688 2690 10.1093/bioinformatics/btl446 16928733

[B103] Altschul SF Madeen TL Schaffer AA Zhang J Zhang Z Miller W Lipman DJ Gapped BLAST and PSI-BLAST: a new generation of protein database search programs Nucleic Acids Res 1997 25 3389 3402 10.1093/nar/25.17.3389 9254694 PMC146917

[B104] Kearney B Staskawicz BJ Widespread distribution and fitness contribution of *Xanthomonas campestris *avirulence gene *avrBs2* Nature 1990 346 385 386 10.1038/346385a0 2374611

[B105] Roden JA Belt B Ross JB Tachibana T Vargas J Mudgett MB A genetic screen to isolate type III effectors translocated into pepper cells during *Xanthomonas *infection Proc Natl Acad Sci USA 2004 101 16624 16629 10.1073/pnas.0407383101 15545602 PMC534543

[B106] Furutani A Takaoka M Sanada H Noguchi Y Oku T Tsuno K Ochiai H Tsuge S Identification of novel type III secretion effectors in *Xanthomonas oryzae *pv. *oryzae* Mol Plant Microbe Interact 2009 22 96 106 10.1094/MPMI-22-1-0096 19061406

[B107] Jiang W Jiang BL Xu RQ Huang JD Wei HY Jiang GF Cen WJ Liu J Ge YY Li GH Su LL Hang XH Tang DJ Lu GT Feng JX He YQ Tang JL Identification of six type III effector genes with PIP box in *Xanthomonas campestris *pv. *campestris *and five of them contribute individually to full pathogenicity Mol Plant Microbe Interact 2009 22 1401 1411 10.1094/MPMI-22-11-1401 19810809

[B108] Kim JG Li X Roden JA Taylor KW Aakre CD Su B Lalonde S Kirik A Chen Y Baranage G McLane H Martin GB Mudgett MB *Xanthomonas *T3S effector XopN suppresses PAMP-triggered immunity and interacts with a tomato atypical receptor-like kinase and TFT1 Plant Cell 2009 21 1305 1323 10.1105/tpc.108.063123 19366901 PMC2685636

[B109] Metz M Dahlbeck D Morales CQ Al Sady B Clark ET Staskawicz BJ The conserved *Xanthomonas campestris *pv. *vesicatoria *effector protein XopX is a virulence factor and suppresses host defense in *Nicotiana benthamiana* Plant J 2005 41 801 814 10.1111/j.1365-313X.2005.02338.x 15743446

[B110] Guidot A Prior P Schoenfeld J Carrere S Genin S Boucher C Genomic structure and phylogeny of the plant pathogen *Ralstonia solanacearum *from gene distribution analysis J Bacteriol 2007 189 377 387 10.1128/JB.00999-06 17085551 PMC1797399

[B111] Park DS Hyun JW Park YJ Kim JS Kang HW Hahn JH Go SJ Sensitive and specific detection of *Xanthomonas axonopodis *pv. *citri *by PCR using pathovar specific primers based on HrpW gene sequences Microbiol Res 2006 161 145 149 10.1016/j.micres.2005.07.005 16427518

[B112] Xu RQ Li XZ Wei HY Jiang B Li K He YQ Feng JX Tang JL Regulation of eight *avr *genes by *hrpG *and *hrpX *in *Xanthomonas campestris *pv. *campestris *and their role in pathogenicity Progress in Natural Science 2006 16 1288 1294 10.1080/10020070612330014

[B113] Noel L Thieme F Nennstiel D Bonas U C-DNA-AFLP analysis unravels a genome-wide *hrpG*-regulon in the plant pathogen *Xanthomonas campestris *pv. *vesicatoria* Mol Microbiol 2001 41 1271 1281 10.1046/j.1365-2958.2001.02567.x 11580833

[B114] Thieme F Szczesny R Urban A Kirchner O Hause G Bonas U New type III effectors from *Xanthomonas campestris *pv. *vesicatoria *trigger plant reactions dependent on a conserved N-myristoylation motif Mol Plant Microbe Interact 2007 20 1250 1261 10.1094/MPMI-20-10-1250 17918627

[B115] Thieme F Genombasierte Identifizierung neuer potentieller Virulenzfaktoren von *Xanthomonas campestris *pv. *vesicatoria* Thesis 2008 Mathematisch-Naturwissenschaftlich-Technische Fakultät der Martin-Luther Universität, Halle-Wittenberg

