# Supplementary material for: Comparative genomics reveals diversity among xanthomonads infecting tomato and pepper
Source: BMC Genomics. 2011 Mar 11;12:146. doi: 10.1186/1471-2164-12-146 (PMC3071791; doi:10.1186/1471-2164-12-146)
Supplement: Additional file 1 — Table S1: General features of the sequencing data and of the de novo assemblies of draft genomes of Xv, Xp and Xg using individual sequencing methods. [file 1471-2164-12-146-S1.DOC]

**Additional file 1** – Table S1: General features of the sequencing data and of the *de novo* assemblies of draft genomes of *Xv*, *Xp* and *Xg* using individual sequencing methods.

|  | *Xanthomonas vesicatoria* (*Xv*) | *Xanthomonas perforans* (*Xp*) | *Xanthomonas gardneri* (Xg) |
| --- | --- | --- | --- |
| *De novo* assembly based on 454 data | | | |
| Number of reads | 397,923 | 476,897 | 408,422 |
| Number of bases | 39,726,904 | 45,692,099 | 38,828,836 |
| Mean length of reads | 99.84 | 95.81 | 95.07 |
| Max length of reads | 351 | 240 | 239 |
| Min of length of reads | 35 | 35 | 35 |
| Number of assembled contigs | 4,181 | 2,360 | 4,540 |
| Mean length of contigs | 1,235 | 2,036 | 1,060 |
| N50 | 701 | 317 | 767 |
| Length of the longest contig | 17,234 | 38,931 | 32,542 |
| Total length of contigs | 5,167,430 | 4,805,047 | 4,816,042 |
| Calculated genome coverage | 7.78X | 8.95X | 7.61X |
|  |  |  |  |
| *De novo* assembly based on Solexa data | | | |
| Number of reads | 10,567,052 | 7,470,680 | 8,463,426 |
| Number of bases | 608,552,220 | 448,240,800 | 507,805,560 |
| Max length of reads | 60 | 60 | 60 |
| Min of length of reads | 60 | 60 | 60 |
| Number of assembled reads | 10,142,537 | 7,150,918 | 8,184,012 |
| Number of assembled contigs | 568 | 492 | 722 |
| Mean length of contigs | 9,275 | 9,866 | 7,261 |
| N50 | 84 | 68 | 112 |
| Length of the longest contig | 69,349 | 78,549 | 82,425 |
| Total length of contigs | 5,268,663 | 4,854,240 | 5,242,611 |
| Calculated genome coverage | 115X | 88X | 94X |
