# Supplementary material for: Comparative genomics reveals diversity among xanthomonads infecting tomato and pepper
Source: BMC Genomics. 2011 Mar 11;12:146. doi: 10.1186/1471-2164-12-146 (PMC3071791; doi:10.1186/1471-2164-12-146)
Supplement: Additional file 2 — Figure S2: 1a) Phylogenetic tree based on MUMi indices; 1b) Distance matrix based on MUMi indices. MUMi program was used to calculate pairwise distances between draft genomes and reference Xanthomonas genomes. [file 1471-2164-12-146-S2.PDF]

Additional file 2 – Figure S2: 1a) Phylogenetic tree based on MUMi indices; 1b) Distance matrix based on MUMi indices. MUMi program was used to calculate pairwise distances between draft genomes and reference *Xanthomonas* genomes.

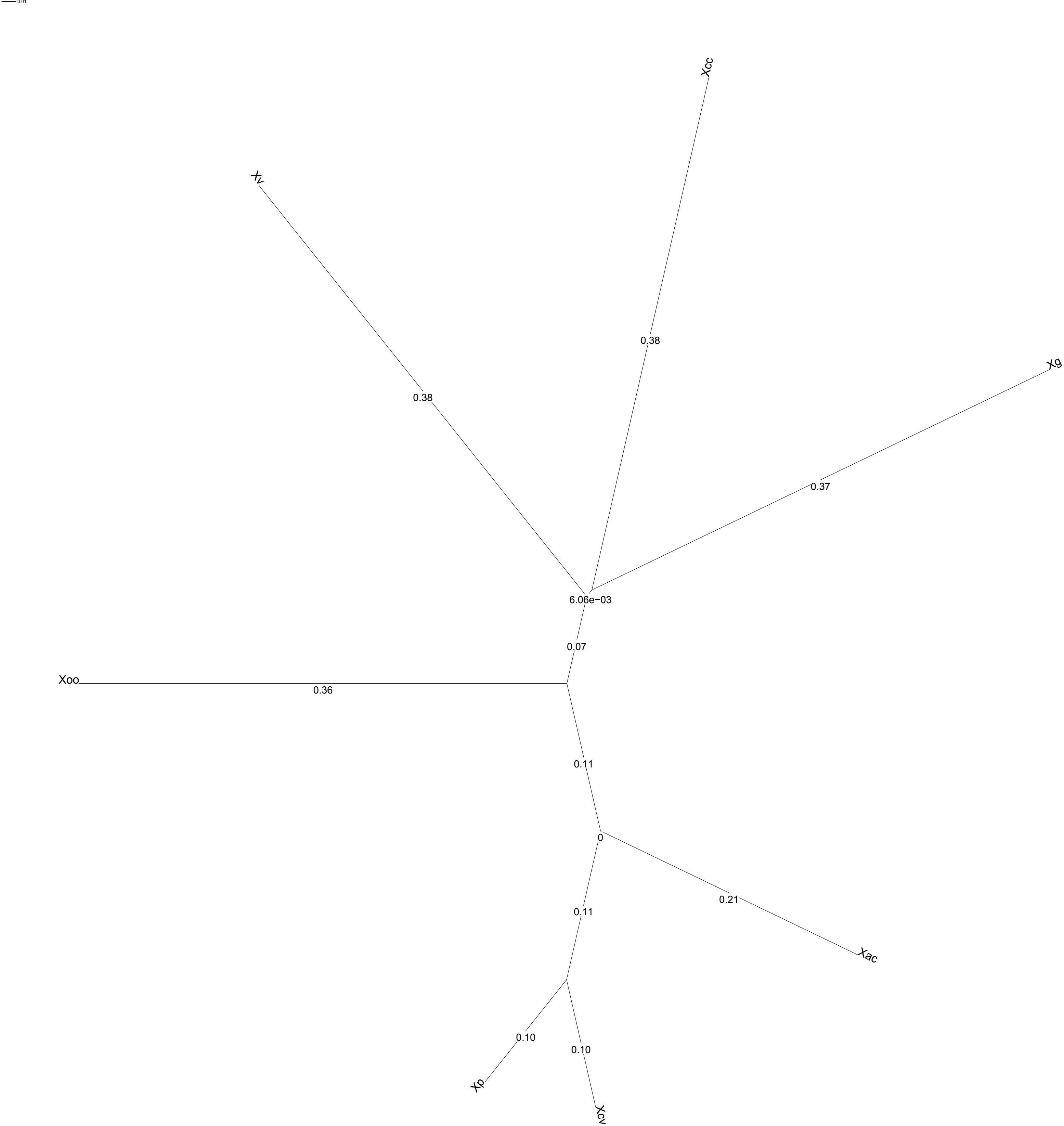
0.01
Xcc
Xv
Xg
0.38
0.38
0.37
6.06e−03
0.07
Xoo
0.36
0.11
0
0.21
0.11
Xac
0.10
0.10
Xp
Xcv

	Xac	Xcc	Xoo	Xcv	Xv	Xp	Xg
Xac	0	0.77	0.67	0.41	0.76	0.41	0.75
Xcc	0.77	0	0.81	0.77	0.77	0.77	0.75
Xoo	0.67	0.81	0	0.67	0.80	0.67	0.79
Xcv	0.41	0.77	0.67	0	0.76	0.19	0.76
Xv	0.76	0.77	0.80	0.76	0	0.76	0.76
Xp	0.41	0.77	0.67	0.19	0.76	0	0.76
Xg	0.75	0.75	0.79	0.76	0.76	0.76	0
