# Supplementary material for: Comparative genomics reveals diversity among xanthomonads infecting tomato and pepper
Source: BMC Genomics. 2011 Mar 11;12:146. doi: 10.1186/1471-2164-12-146 (PMC3071791; doi:10.1186/1471-2164-12-146)
Supplement: Additional file 3 — Table S3: Whole genome comparisons using MUMmer dnadiff program. % coverage of the aligned contigs and % identities of the respective contigs against reference genomes has been shown for each draft genome. [file 1471-2164-12-146-S3.DOC]

**Additional file 3** – Table S3: Whole genome comparisons using MUMmer dnadiff program. % coverage of the aligned contigs and % identities of the respective contigs against reference genomes has been shown for each draft genome.

| **Genome comparison** | | **% of contigs of draft genome aligned** | **% of average identity for the aligned sequences** |
| --- | --- | --- | --- |
| Xp | Xcv | 85.57 | 98.1 |
|  | Xac | 85.91 | 93.8 |
|  | Xcc | 74.23 | 87.36 |
|  | Xoo MAFF | 77.32 | 90.5 |
| Xg | Xcv | 78.44 | 88.57 |
|  | Xac | 79.71 | 88.05 |
|  | Xcc | 83.33 | 88.83 |
|  | Xoo MAFF | 72.83 | 87.9 |
| Xv | Xcv | 83.11 | 87.86 |
|  | Xcc | 76.35 | 87.37 |
|  | Xac | 80.07 | 87.90 |
|  | Xoo MAFF | 69.26 | 87.68 |
