# Supplementary figures and images for: Comparative genomics reveals diversity among xanthomonads infecting tomato and pepper
Source: BMC Genomics. 2011 Mar 11;12:146. doi: 10.1186/1471-2164-12-146 (PMC3071791; doi:10.1186/1471-2164-12-146)

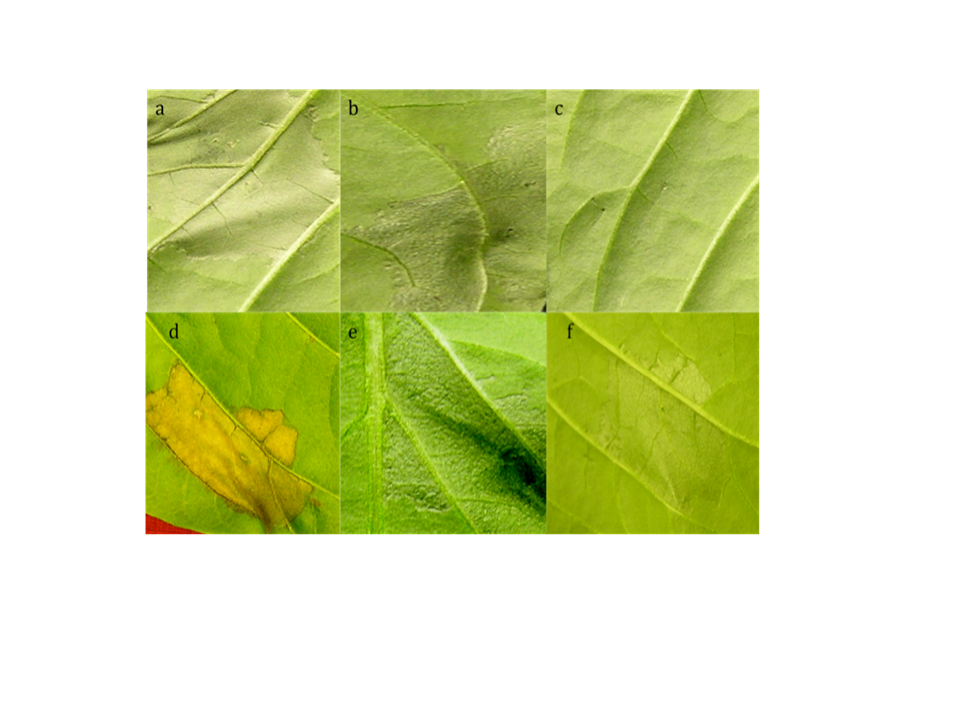

Supplement: Additional file 4 — Figure S4: AvrBs2-based HR assay confirms translocation of novel effectors. Hypersensitive response reaction indicating presence of translocation signal was recorded 24 hrs after inoculation on pepper cv. ECW20R with candidate effectors xopZ2 (a), avrBs1 (b), xopG (d), xopAM (e), xopAO (f) conjugated in race 6 strain along with control race 6 strain (c). All the strains showed water-soaking on pepper cv. ECW after 48 hrs after inoculation [file 1471-2164-12-146-S4.TIFF]
