# Supplementary material for: Comparative genomics reveals diversity among xanthomonads infecting tomato and pepper
Source: BMC Genomics. 2011 Mar 11;12:146. doi: 10.1186/1471-2164-12-146 (PMC3071791; doi:10.1186/1471-2164-12-146)
Supplement: Additional file 5 — Table S5: Evidence of the horizontal gene transfer using Alien_hunter analysis. [file 1471-2164-12-146-S5.DOC]

**Additional file 5** – Table S5: Evidence of the horizontal gene transfer using alien hunter analysis.

| Gene/ Gene cluster | Locus tag | Score for Alien Hunter (Threshold = 12.496) | % GC | tRNA/transposase/mobile genetic elements in the vicinity | Evidence of HGT |
| --- | --- | --- | --- | --- | --- |
| avrBs1 | XGA_0724 | 32.031 | 45 | Transposase | Good |
| xopAO | XGA_1250 | 13.735 | 48 | Predicted to be located on plasmid | Good |
| xopAS | XGA_0764/XGA_0765 | 19.844 | 59 | Transposase | Good |
| xopG | XGA_4501  XVE_4777  XCV1298 | 21.272 | 50 | ISxac2 transposase in *Xcv* | Good |
| xopAQ | XGA_2091 | Does not belong to anomalous region | 51 | Could not be predicted | Weak |
| xopZ2 | XGA_2762  XVE_3221 | Does not belong to anomalous region | 69 | IS30 transposase 3000 bps apart | Weak |
| LPS cluster | XPE_3787 to XPE_3795 | Belongs to anomalous region | 50 | No | Good |
| Bacteriocin cluster | XPE_0786 to XPE_0790 | Belongs to anomalous region | 50 | tranposase | Good |
