# Supplementary material for: Comparative genomics reveals diversity among xanthomonads infecting tomato and pepper
Source: BMC Genomics. 2011 Mar 11;12:146. doi: 10.1186/1471-2164-12-146 (PMC3071791; doi:10.1186/1471-2164-12-146)
Supplement: Additional file 6 — Table S6: Genes/contigs representing T6SS in draft genomes as compared to Xcv. [file 1471-2164-12-146-S6.DOC]

**Additional file 6** – Table S6: Genes/contigs representing T6SS in draft genomes as compared to *Xcv*.

| **T6SS subtype #1** | | **T6SS subtype #3** | | | |
| --- | --- | --- | --- | --- | --- |
| Xp | XCV homologs | Xp | XCV homologs | Xv | XCV homologs |
| Contig 33 | XCV2120-XCV2127(N) | Contig 120 | XCV4244-XCV4236(N) | Contig 233 | XCV4244-XCV4216 |
| Contig 287 | XCV2127(i) | Contig 287 | XCV4236(i) |  |  |
| Contig 288 | XCV2127(i) | Contig 288 | XCV4236(i) |  |  |
| Contig 291 | XCV2127(i) | Contig 291 | XCV4236(i) |  |  |
| Contig 238 | XCV2127(i) | Contig 238 | XCV4236(i) |  |  |
| Contig 254 | XCV2127(i) | Contig 254 | XCV4236(i) |  |  |
| Contig 90 | XCV2127(C)-XCV2137(N) | Contig 44 | XCV4236(C)-XCV4216 |  |  |
| Contig 240 | XCV2137(C)-XCV2144 | no homolog | XCV4215 | no homolog | XCV4215 |
|  |  | Contig 116 | XCV4214-XCV4209(N) | Contig 183 | XCV4214-XCV4212(N) |
|  |  | Contig 133 | XCV4209(C)-XCV4206(N) | Contig 148 | XCV4213(N)-XCV4206(N) |
|  |  | Contig 233 | XCV4206(i) |  |  |
|  |  | Contig 195 | XCV4206(C) | Contig 175 | XCV4206(C) |
