# Supplementary material for: Comparative genomics reveals diversity among xanthomonads infecting tomato and pepper
Source: BMC Genomics. 2011 Mar 11;12:146. doi: 10.1186/1471-2164-12-146 (PMC3071791; doi:10.1186/1471-2164-12-146)
Supplement: Additional file 7 — Table S7: Domain architecture and distribution of proteins with HD-GYP, GGDEF and/or EAL domains encoded by genomes of different Xanthomonas strains. [file 1471-2164-12-146-S7.PDF]

Additional file 7 – Table S7. Domain architecture and distribution of proteins with HD-GYP, GGDEF and/or EAL domains encoded by genomes of different *Xanthomonas* strains

Domain architecture of proteins	Xcc8004	Xcc33913	XooKACC10331	XooPXO99A	XooMAFF311018	Xcv	Xv	Xp	Xg
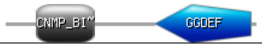	XC0249	XCC0239	absent	absent	absent	XCV0266	0573	1195	1780
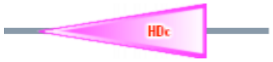	XC0362	XCC0350	XOO4220	ORF03945	XOO3989	XCV0363	3039	4492	3433
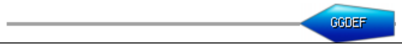	XC0420	XCC0407	XOO0111	ORF03778	XOO0067	XCV0450	2497	2956	1448
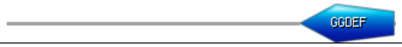	XC0496	XCC0484	XOO0520	ORF02944	XOO0483	XCV0529	1437	3029	2716
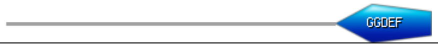	XC0613	XCC3546	absent	absent	absent	XCV0645	2531	4167	4044[1]
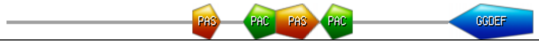	XC0637	XCC3523	XOO4021	ORF04155	XOO3792	XCV0667	2531	3570	5056
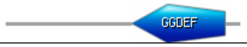	XC0641	XCC3519	XOO4016[b]	ORF04147	XOO3786	XCV0672	0665	3574	4680
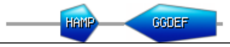	XC0675	XCC3486	XOO3988	ORF04275	XOO3759	XCV0699	1226	4018	4301
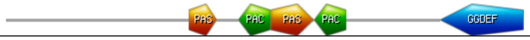	XC0831	XCC3333	XOO0933[a]	ORF02615[a]	XOO0855[a]	XCV3588	1507	1922	2867
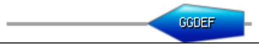	XC1036	XCC3120	absent	absent	absent	absent	absent	absent	absent
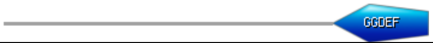	XC1383	XCC2731	XOO1440	ORF02019	XOO1324	XCV3048	5029[2]	1275	0529
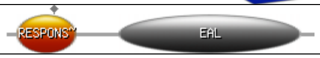	XC1411	XCC2703	XOO1467	ORF04753	XOO1368	XCV3023	2771	0357	5134
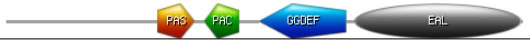	XC1476	XCC2641	XOO1551	ORF04826	XOO1439	XCV2973	3314	1829	1596
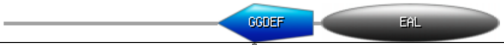	XC1582	XCC2563	XOO3246	ORF01278	XOO3074	XCV2860	1735	0013	2269
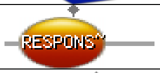	XC1755	XCC2361	XOO02798	ORF00476	XOO2640	XCV2672	0992	0406	1397
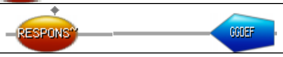	XC1766	XCC2350	XOO2787	ORF00466	XOO2627	XCV2660	0980	0394	2619
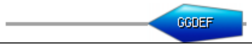	XC1803	XCC2313	absent	absent	absent	XCV2623	0942	3324	5028
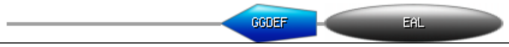	XC1824	XCC2291	XOO2725	ORF00403	XOO2570	XCV2595	3188	3346	3744
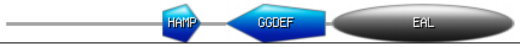	XC1841	XCC2274	XOO2708	ORF00384	XOO2553	XCV2579	3205	1807	2022
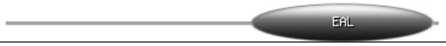	XC2161	XCC2023	absent	absent	absent	XCV2102	1554	2913	3058
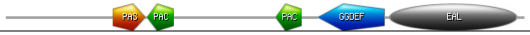	XC2226	XCC1959	XOO2561	ORF01021	XOO2420	XCV2041	4978[3]	3128	1884
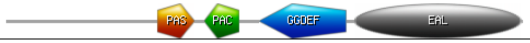	XC2228	XCC1958	XOO2563	ORF01019	XOO2422	XCV2039	4980	3130	1886
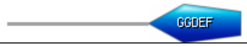	XC2274	XCC1913	XOO2614	ORF00967	XOO2473	XCV1985	4032	1307	1461
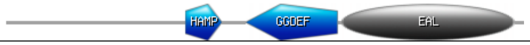	XC2275	XCC1912	XOO2615	ORF00965	XOO2474	XCV1983	4030	1306	1460
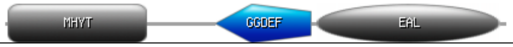	XC2276	XCC1911	XOO2616	ORF00964	XOO2475	XCV1982	absent	1305	1459
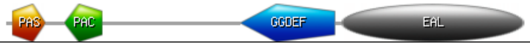	XC2324	XCC1865	XOO2860	ORF00058	XOO2715	XCV1929	2941	0537	0024

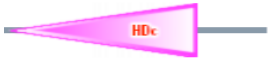	XC2335	XCC1854	XOO2871	ORF00070	XOO2725	XCV1917	2924	0522	2956
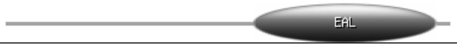	XC2459	XCC1777	XOO2354	ORF00678	XOO2236	XCV1825	5096	0307	3401
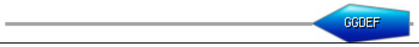	XC2715	XCC1520	absent	absent	absent	XCV1610	3722	1158	2000
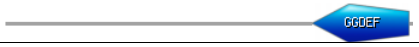	XC2793	XCC1445	XOO2330[a]	ORF00647	XOO2206[a]	XCV1546	0243	3986	1105
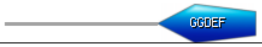	XC2795	XCC1443	absent	ORF00649	XOO2208	XCV1544	0241	3988	1961
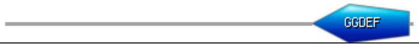	XC2866	XCC1372	absent	absent	absent	XCV1477	171	4823	4622
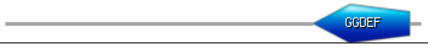	XC2946	XCC1294	XOO1879	ORF01741	XOO1775	XCV1398	2082	4745	1494
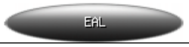	XC3163	XCC1086	absent	absent	absent	XCV1210	1545	221	0886
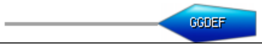	XC3800	XCC3729	absent	absent	absent	absent	absent	absent	absent
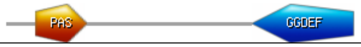	XC3829	XCC3759	absent	absent	absent	absent	absent	absent	absent
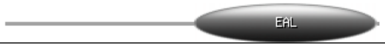	XC3962	XCC3877	XOO4445[a]	ORF03877[a]	XOO4185[a]	XCV4051	0685	2596	0663
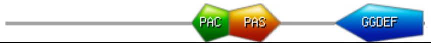	XC4313	XCC4224	absent	ORF04275	absent	XCV4470	4882	4444	4813
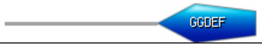	absent	absent	XOO2331	ORF00647	absent	absent	absent	absent	absent
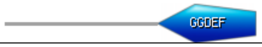	absent	absent	absent	absent	absent	XCV2971	3316	1827	1598
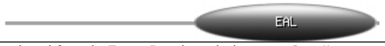	absent	absent	absent	absent	absent	XCVd0150[c]	absent	absent	absent

[1]Domains were predicted using the prosite research tool from the Expert Protein analysis system (http://ca.expasy.org/prosite). This analysis revealed all proteins with the conserved HD domain; the subset of these proteins with the HD-GYP domain was identified by checking for the presence of the GYP motif and by BLASTP alignments. The proteins also contain a variety of input domains including cyclic nucleotide binding domain (CNMP_BI), PAS, PAC, and uncharacterized domains or undefined regions are marked by a /. Complete domain descriptions and functions of other domains are given at http://ca.expasy.org/prosite. [a] Proteins with N-terminal truncations; [b] Gene has an internal stop codon. [c]Plasmid-borne.
