# Supplementary material for: Comparative genomics reveals diversity among xanthomonads infecting tomato and pepper
Source: BMC Genomics. 2011 Mar 11;12:146. doi: 10.1186/1471-2164-12-146 (PMC3071791; doi:10.1186/1471-2164-12-146)
Supplement: Additional file 8 — Table S8: Genes unique to Xp, grouped in clusters. [file 1471-2164-12-146-S8.DOC]

**Additional file 8** – Table S8: Genes unique to *Xp*, grouped in clusters.

| Locus tag in *Xp*/ Gene OID | Distribution of flanking genes | Function |
| --- | --- | --- |
| Cluster 1- LPS cluster genes | | |
| XPE_3787 to XPE_3795 | Present in *Xcv* | Lipopolysaccharide biosynthesis cluster |
| Cluster 2- Chemotaxis protein histidine kinase inactivated by transposase carrying 3 genes (in yellow) along with it in *Xp*. | | |
| XPE_4460 | In all 4 | chemotaxis protein histidine kinase |
| XPE_4461 | In all 4 | transposase |
| XPE_4462 |  | Fe-S oxidoreductase |
| XPE_4463 |  |  |
| XPE_4464 |  |  |
| XPE_4465 | In all 4 | chemotaxis protein histidine kinase |
|  |  |  |
| Cluster 3- Carrying unique genes in *Xp* not present in any plant pathogens | | |
| XPE_1809 |  | Transposase |
| XPE_1810 |  | TIR-like domain, cyclic nucleic acid binding domain |
| XPE_1811 |  | Hypothetical protein |
| XPE_1812 |  | Hypothetical protein |
| XPE_1813 |  | Hypothetical protein |
|  |  |  |
| Cluster 4- *avrXv4* and phage genes in the neighbourhood | | |
|  |  |  |
|  |  |  |
| Cluster 5- *XopC* from *Xcv* is replaced by other unique genes in *Xp* | | |
| XPE_3067 | present in 306 | hypothetical protein |
| XPE_3068 | present in 306 | hypothetical protein |
| XPE_3069 | XAC2120 |  |
| XPE_3070 | mdmC from Xac306 | Predicted O-methyltransferase |
| XPE_3071 | not called in gene calling in 306 | Hypothetical protein |
|  |  |  |
| Cluster 6- Carrying bacteriocin genes | | |
| XPE_0786 to XPE_0790 |  |  |
|  |  |  |
| Cluster 7- flanked by phage integrase | | |
| XPE_2401 |  | Predicted transcription regulator containing HTH domain |
| XPE_2402 |  | Uncharacterized protein conserved in bacteria |
| XPE_2403 | present in *Xv* |  |
| XPE_2404 |  |  |
| XPE_3894 |  | plasmid mobilization system relaxase |
| XPE_3895 | XCV1122 |  |
| XPE_3896 | 52% hypothetical protein [Legionella pneumophila str. Corby] | predicted ATPase |
| XPE_3897 |  | hypo protein from Legionella |
| XPE_3898 | present in *Xv, Xg* |  |
| XPE_3899 | in *Xv*, XCV1116 |  |
| XPE_3900 | *Xcc*B100_3109 | exonuclease VII |
| XPE_3901 | present in *Xg* |  |
|  |  |  |
| Cluster 8- Upstream flanking genes are conserved in order in all xanthomonads; while downstream are transposase genes in *Xcv.* | | |
| XPE_3601 | present in all xanthomonads |  |
| XPE_3602 |  |  |
| XPE_3603 |  |  |
| XPE_3604 |  |  |
| XPE_3605 |  |  |
| XPE_3606 |  |  |
| XPE_3607 |  |  |
| XPE_3608 |  |  |
| XPE_3609 |  |  |
|  |  |  |
| cluster 9- Flanking genes conserved in *Xcv* | | |
| XPE_3366 | XAUB_37550 95% | hypothetical protein |
| XPE_3367 | XCV0352 |  |
| XPE_3368 | XCV0353 |  |
| XPE_3369 | no hit to any plant pathogen | hypothetical protein |
| XPE_3370 | no hit to any plant pathogen | hypothetical protein |
| XPE_3371 | no hit to any plant pathogen | hypothetical protein |
| XPE_3372 | no hit to any plant pathogen | hypothetical protein |
| XPE_3373 | no hit to any plant pathogen | Activator of Hsp90 ATPase homolog 1-like protein. |
| XPE_3374 | no hit to any other plant pathogen | hypothetical protein |
|  |  |  |
| Cluster 10- Upstream and downstream flanking genes present in *Xcv* | | |
| XPE_1376 | no hit to any other plant pathogen |  |
| XPE_1377 | No hit to any other plant pathogen |  |
| XPE_1378 | no hit to any other plant pathogen |  |
| XPE_1379 | no hit to any other plant pathogen |  |
| XPE_1380 |  | EF-hand calcium binding protein |
|  |  |  |
| Cluster 11- Upstream, downstream flanking genes also present in *Xcv* | | |
| XPE_0135 | XAC3183 | Hypothetical protein |
| XPE_0136 | XAC3182 | Hypothetical protein |
|  |  |  |
| Cluster 12- Flanking genes present in Xcv. In place of following unique genes, Xcv contains ISXac3 transposase. | | |
| XPE_0734 |  | signal peptide, transmemb helices |
| XPE_0735 |  | hypothetical protein |
| XPE_0736 | Xccb100_0356, vasculorum and musacearum | hypothetical protein |
|  |  |  |
| Cluster 13 | | |
| XPE_2183 | In Xoo | Type I site-specific restriction-modification system, R (restriction) subunit and related helicases |
| XPE_2185 | Xoo | Type I restriction-modification system methyltransferase subunit |
| XPE_2187 | Xoo | Uncharacterized conserved protein |
| XPE_2190 | Xoo | Uncharacterized conserved protein |
| XPE_2192 | Xoo | Type I restriction-modification system methyltransferase subunit |
| XPE_2194 | Xoo | Type I site-specific restriction-modification system, R (restriction) subunit and related helicases |
| XPE_2195 | Xoo, Xoc | hypothetical protein |
