# Supplementary material for: Comparative genomics reveals diversity among xanthomonads infecting tomato and pepper
Source: BMC Genomics. 2011 Mar 11;12:146. doi: 10.1186/1471-2164-12-146 (PMC3071791; doi:10.1186/1471-2164-12-146)
Supplement: Additional file 9 — Table S9: Genes common to all pepper pathogens but absent from Xp. [file 1471-2164-12-146-S9.DOC]

**Additional file 9** – Table S9: Genes common to all pepper pathogens but absent from *Xp*.

| Locus tag in Xcv85-10 | Gene symbol | Product name | Evidence to be involved in pathogenicity/ virulence |
| --- | --- | --- | --- |
| Genes with the known functions | | | |
| XCV2278 |  | Pectate lyase precursor |  |
| XCV3713 | wxcL | Glycosyltransferase |  |
| XCV3715 | wxcN | Putative membrane protein involved in synthesis of cell surface polysaccharide |  |
| XCV3716 | wxcO | Putative carbohydrate translocase | Alavi, SM *et. al*., 2008 in *X. fuscans* – bean pathosystem and this study. |
| XCV3718 | gmd | GDP-mannose 4,6-dehydratase (EC: 4.2.1.47) |  |
| XCV3720 | wxcB | Putative protein kinase |  |
| XCV3722 | wzm | O-antigen ABC transporter permease |  |
| XCV4257 | rpmB | LSU ribosomal protein L28P |  |
| XCV1298 |  | Type III effector (homolog of hopH1 from *Pseudomonas syringae*) |  |
| XCV1839 |  | Hypothetical protein | This study |
| XCVc0007 | kfrA | KfrA protein |  |
| XCV0510 | hsdS1 | Type I site-specific deoxyribonuclease (specificity subunit) |  |
| XCV0513 | hsdM1 | Type I site-specific deoxyribonuclease (modification subunit) |  |
| XCV2820 |  | Putative type IV pilus assembly protein PilV |  |
| XCV3312 |  | Transcriptional regulator, AraC family |  |
| XCV2191 |  | Putative DoxD-like family membrane protein |  |
|  |  |  |  |
| Genes coding for mobile genetic elements | | | |
| XCVb0012 |  | Putative ISxac3 transposase (fragment) |  |
| XCVb0018 | tnpR | Tn5045 resolvase |  |
| XCVc0040 |  | Site-specific recombinase/resolvase family protein |  |
| XCVd0025 |  | ISxac3 transposase (fragment) |  |
| XCVd0071 |  | Phage integrase family protein |  |
| XCVd0097 | tnpA | Tn5044 transposase |  |
| XCVd0109 | tnpR | Tn5045 resolvase |  |
| XCVd0115 |  | Tn5044 traposase |  |
| XCV0355 |  | ISxac3 transposase |  |
| XCV0619 |  | Transposase |  |
| XCV0706 |  | ISxac3 transposase |  |
| XCV1118 |  | ISxac3 transposase |  |
| XCV1553 |  | Phage-related integrase |  |
| XCV1698 |  | ISxac3 transposase |  |
| XCV1843 |  | ISxac3 transposase |  |
| XCV1848 |  | Putative integrase/recombinase |  |
| XCV2158 |  | ISxac3 transposase |  |
| XCV2217 |  | Phage-related integrase |  |
| XCV2261 |  | Phage-related integrase |  |
| XCV2263 |  | ISxac3 transposase (fragment) |  |
| XCV2273 |  | Tn5044 transposase |  |
| XCV2295 |  | Putative ISxac3 transposase (fragment) |  |
| XCV2439 |  | Tn5044 trasposase |  |
| XCV2453 |  | Filamentous phage Cf1c protein |  |
| XCV2461 |  | Filamentous phage phiLf related protein |  |
| XCV2474 |  | Filamentous phage Cf1c protein |  |
| XCV2477 |  | ISXac3 transposase |  |
| XCV2484 |  | Phage-related integrase |  |
| XCV2615 |  | Integrase |  |
| XCV2690 |  | ISxac3 transposase |  |
| XCV2712 |  | Putative transposase (fragment) |  |
| XCV2867 |  | ISxac3 trasposase |  |
| XCV3384 |  | ISxac3 trasposase |  |
| XCV3397 |  | ISxac3 trasposase |  |
| XCV3410 |  | ISxac3 trasposase |  |
|  |  |  |  |
| Genes with function unknown | | | |
| XCVd0055 |  |  |  |
| XCV0648 |  |  |  |
| XCV1188 |  |  |  |
| XCV1189 |  |  |  |
| XCV1187 |  |  |  |
| XCV1303 |  |  |  |
| XCV1596 |  |  |  |
| XCV1937 |  |  |  |
| XCV2455 |  |  |  |
| XCV2857 |  |  |  |
| XCV2958 |  |  |  |
| XCV3162 |  |  |  |
| XCV3326 |  |  |  |
| XCV3986 |  |  |  |
| XCV4135 |  |  |  |
| XCV4262 |  |  |  |
| XCV4421 |  |  |  |
